# The filopodial myosin DdMyo7 is a slow, calcium-regulated motor

**DOI:** 10.1016/j.jbc.2025.108371

**Published:** 2025-03-03

**Authors:** Casey Eddington, Margaret A. Titus

**Affiliations:** 1 Department of Genetics, Cell Biology, and Development, University of Minnesota, Minneapolis, Minnesota, USA; 2 Graduate Program in Biochemistry, Molecular Biology, and Biophysics, University of Minnesota, Minneapolis, Minnesota, USA

**Keywords:** DdMyo7, MyTH4-FERM myosin, unconventional myosin, molecular motor, actin cytoskeleton, filopodia

## Abstract

MyTH4-FERM (MF) myosins are a family of molecular motors with critical roles in the formation and organization of thin membrane protrusions supported by parallel bundles of actin - filopodia, microvilli, and stereocilia. The amoeboid MF myosin DdMyo7 is essential for filopodia formation but its mechanism of action is unknown. The motor properties of a forced-dimer of the DdMyo7 motor were characterized using an *in vitro* motility assay to address this question. The DdMyo7 motor associates with two different light chains, the *Dictyostelium* calmodulins CalA and CalB, whose binding is shown to be sensitive to the presence of calcium. Total internal reflection fluorescence motility assays of the dimerized DdMyo7 motor reveal that it is a slow, processive motor that moves along actin at ∼ 40 nm/sec, and the activity of the motor is significantly reduced in the presence of Ca^2+^. The speed of DdMyo7 is similar to that of other Myo7 family members such as human Myo7A and fly DmMyo7A, but is at least 10-fold slower than the mammalian filopodial MF myosin, Myo10. The results show that evolutionarily distant native filopodial myosins can promote filopodia elongation using motors with distinct properties, revealing diverse mechanisms of myosin-based filopodia formation.

Myosins are actin-based motor proteins that play important roles in a diverse array of cellular functions, serving as force generators, anchors, and macromolecular transporters. The MyTH4-FERM (MF; myosin tail homology—Band 4.1, Ezrin, Radixin, and Moesin) family of myosins, Myo7, Myo10, and Myo15, are critical for forming or organizing membrane protrusions supported by parallel bundles of actin, filopodia, microvilli, and stereocilia (1). These specialized protrusions are important for cellular exploration (filopodia), formation of a barrier along the intestinal epithelium (microvilli), and hearing/balance (stereocilia). MF myosins are characterized by the presence of one or two signature MF domains, protein interaction modules, in their C-terminal tail domains. These myosins not only move along or anchor actin filaments but they also have roles in building filopodia (Myo7 in amoebae and Myo10 in mammalian cells) (2, 3, 4) or stereocilia (Myo15A) (5, 6). Their activity is controlled by calmodulin (CaM) or CaM-related light chains that bind to IQ motifs in the neck or by head–tail autoinhibition (1, 7). In the case of Myo7 and Myo10, these motors operate as dimers (8, 9, 10). Myo7 appears to dimerize by partner binding and Myo10 by a dimerization region that follows the lever arm (3, 9, 10). In the case of Myo10, it is an antiparallel dimer that is well-suited for moving efficiently along parallel bundles of the actin core of filopodia (9). It is not known if Myo7 forms a parallel or antiparallel dimer. While the contribution of myosins to filopodia formation and stereocilia elongation is well-established, their exact role in these processes is not fully understood.

Two models for the role of MF myosins in filopodia formation in mammalian cells have emerged based on studies of Myo10. One envisions a role for the dimerized motor in the convergence of actin filaments at the plasma membrane, aiding in organization of actin filament into parallel bundles and elongation in collaboration with actin polymerases such as VASP (11). In this case, Myo10 may also transport VASP along the growing filopodia to the filopodia tip to promote ongoing polymerization and extension (12, 13). A second model proposes that force generation by the membrane-bound motor at the filopodia tip reduces membrane tension and this is the key for promoting extension (14). This activity is not unique to Myo10, ectopic expression of other motors such as Myo15A, Myo3A, that are docked to the membrane can also result in filopodial elongation (15). Interestingly, not all membrane-bound myosin motors can promote filopodia formation, and it is thought that this may be due to slower velocities (Myo1A and Myo7A), lack of processivity (nonmuscle Myo2A) or some form of mechanosensitivity (Myo5B).

Filopodia extension rates can vary depending on cell type (14, 16, 17, 18). Filopodia extended by mouse root ganglion growth cones and *Drosophila* primary neurons reportedly extend at a rate of 55 nm/s and 87 nm/s, respectively (16, 18), and those of HeLa cells reportedly grow at similar speeds (36 nm/s) (14). In the case of mammalian cell filopodia, their extension rates are significantly slower than that of the dimerized Myo10 motor that moves optimally along bundled actin filaments at 660 nm/sec (9). In contrast, the candidate fly filopodial motor DmMyo7A is slow, walking on actin filaments at an average rate of 7.7 nm/s (3). These observations suggest that the motor activities of Myo10 and DmMyo7A during filopodia formation are harnessed in different ways that remain to be understood. Highly motile *Dictyostelium* cells rapidly extend filopodia at a rate of 400 nm/s (17). Therefore, if the amoeboid filopodia myosin, DdMyo7, transports actin polymerases from the base to the tips of filopodia during elongation (as proposed for Myo10), its velocity on actin filaments might be expected to exceed that of filopodia polymerization/extension. The DdMyo7 motor is uncharacterized, thus, determining its motor properties is critical for understanding the role of this motor in filopodia formation.

## Results

### Identification of the DdMyo7 light chains

Myosin light chains (LCs) play a critical role in stabilizing the neck (LC binding region) during force generation and regulating motor activity. Given the important role that these subunits play in motor activity it is critically important to identify the LCs used by a myosin of interest. The DdMyo7 LCs are unknown, so a critical first step needed for characterizing the DdMyo7 motor was to identify its LCs.

**Figure 1:**
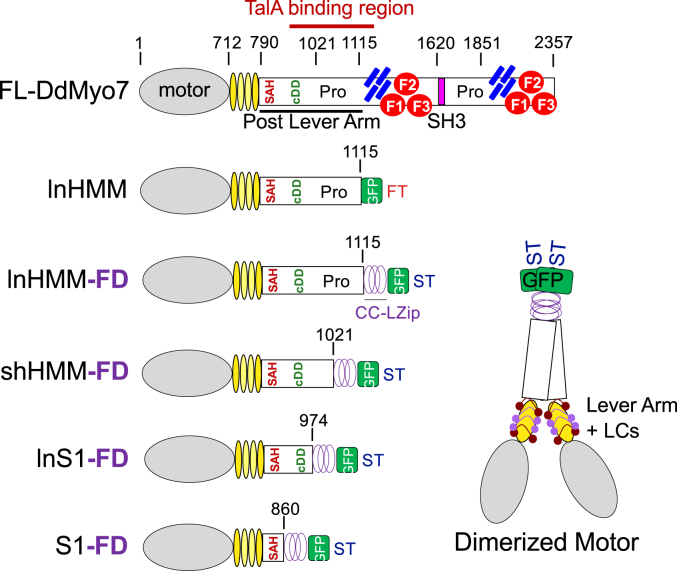
**DdMyo7 motors used in study.** Illustrations of full-length *Dictyostelium.* Myosin 7 (FL-DdMyo7) and motors of varying lengths used in this study. These include long HMM (lnHMM) and forced-dimers of long HMM (lnHMM-FD), short HMM (shHMM-FD), long S1 (lnS1-FD), and S1 (S1-FD). The domains depicted are the motor (*gray oval*), IQ motifs (*yellow ovals*), single alpha-helix (SAH), candidate dimerization domain (cDD), proline-rich regions (Pro), SH3 domain (*purple rectangle*) and MF domains (*blue rods*–MyTH4; *red circles* indicating three lobes of the FERM domain). The GFP (*green square*), dimerization sequence (CC-Lzip *purple* helix) and epitope tags (FT – FLAG Tag; ST – Strep-tag) are also shown. The drawing on the right illustrates a forced-dimer with bound LCs (*purple lines*/*dots*). The previously mapped TalA binding region (amino acids 902–1156) (20) is highlighted in *red* above FL-DdMyo7. HMM, heavy meromyosin; LC, light chain; S1, subfragment 1.

**Figure 2:**
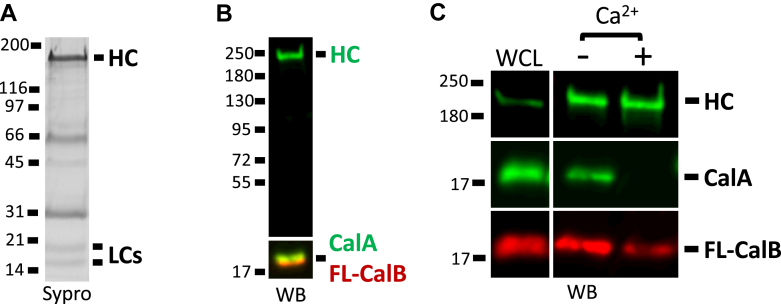
**CalA and CalB are the DdMyo7 light chains and binding is calcium dependent.***A*, DdMyo7 heavy chain (HC) pull downs revealing CalA and CalB as the DdMyo7 light chains (LCs). Following densitometry of the gel lanes, the molar ratios were calculated by normalizing HC, CalA, and CalB signal to their MWs (HC: 36.1, CalA: 83.0, CalB: 83.5, arbitrary units) then determining a ratio of CalA/HC and CalB/HC. CalA/HC: 2.08 ± 0.12, CalB/HC: 2.01 ± 0.16; N = 3. Average LC/HC ratio and S.E.M. reported. *B*, immunoblot of a shHMM-FD pull down probed for shHMM-FD-GFP (HC), CalA, and Flag (FL)-CalB. *C*, DdMyo7 shHMM-FD pull downs in the presence or absence of Ca^2+^. Immunoblots were probed for shHMM-FD-GFP (HC), CalA, and FL-CalB. Immunoblot signal in the presence or absence of Ca^2+^ was normalized to correct for gel load and heavy chain abundance. CalA reduction + Ca^2+^: 99.7% ± 0.15, CalB reduction + Ca^2+^: 75.1% ± 3.46; N = 3. Average reduction percentage and S.E.M. reported. FD, forced-dimer.

A FLAG-tagged motor domain that includes the four LC-binding IQ motifs (long heavy meromyosin, lnHMM, Fig. 1) was ectopically expressed in Talin A (TalA) null cells and then immunoisolated. TalA is a critical cytoskeletal linker protein that links adhesion receptors to the actin cytoskeleton and is a DdMyo7 binding partner that modulates its association with the leading edge of migrating cells (19). TalA robustly binds DdMyo7 near the proline-rich (Pro) region in the DdMyo7 post lever arm (PLA) (Fig. 1) (20). Thus, to avoid isolating a DdMyo7:TalA:F-actin macrocomplex that could contain additional proteins that do not bind DdMyo7, the motor was expressed in *talA* null cells (21). Two low-molecular weight bands were seen in the immunoprecipitation (IP) pellet, indicating the DdMyo7 neck could potentially bind two different LCs (Fig. 2*A*). Two notable bands running at ∼ 60 kD and ∼30 kD are quite likely the mouse IgG1 heavy chain (HC) and LC from the immunoprecipitating antibody (anti-FLAG M2) while a third, fainter band at 45 kD is *Dictyostelium* actin that is frequently found to coprecipitate with the dimerized motor even in the presence of mM concentrations of MgATP. Because CaM is typically the LC for unconventional myosins (7), the motor pull downs were screened for the presence of *Dictyostelium* CaM (CalA) *via* immunoblotting. This revealed CalA to be a DdMyo7 LC, running above the 17 kDa protein standard, consistent with it being the upper band (Fig. 2*B*). The identity of the lower band was determined by mass spectrometry analysis. This revealed the lower band to be *Dictyostelium* CaM-like protein (CalB; Table S1), a small EF-hand protein highly similar to CalA (22). Inspection of the CalB sequence reveals that two of the EF-hands have charge differences in one of the residues critical for Ca^2+^-coordination and two of them are shorter by one amino acid (Fig. S1), suggesting that binding to the IQ motifs might not be Ca^2+^-sensitive. The stoichiometry of the associated LCs was determined by densitometry of a gel of IP pellets stained with SYPRO. A ratio of ∼2:2:1 CalA:CalB:lnHMM, was found, revealing that the four IQ neck is occupied by 2x CalA and 2x CalB molecules, simultaneously (Fig. 2*A*). It should be noted that CalA (17.2 kDa) and CalB (16.8 kDa) are nearly the same molecular weight and would normally be expected to comigrate. However, under the pull-down conditions used here, CalA and CalB are consistently seen to run as two distinct bands (Fig. 2*A*). Similarly, purified CalA and CalB were sometimes observed running separately and sometimes observed running together (Fig. S2). The basis for this inconsistent behavior is currently unknown.

The identity of CalB as a DdMyo7 LC was confirmed by coexpressing a FLAG-CalB (FL-CalB) construct with a dimerized DdMyo7 motor (short heavy meromyosin-forced-dimer-GFP, shHMM-FD-GFP) in *talA* null cells. shHMM-FD-GFP was isolated with GFP trap resin and then probed for FL-CalB using an α-FLAG antibody, confirming that FL-CalB is pulled down with the DdMyo7 motor (Fig. 2*B*). The cellular localization of CalB was also determined by coexpressing GFP-CalB and DdMyo7-mCherry in DdMyo7 null cells. A large cytosolic pool of GFP-CalB was observed and the tagged LC is also seen to brightly colocalize to filopodia tips with DdMyo7-mCherry (Fig. S3*A*). Line scans along the length of filopodia show that the mean GFP intensity (GFP-CalB) peaks along with the mean mCherry intensity (DdMyo7-mCherry) at the filopodium tip (Fig. S3*B*). Together, these results establish that CalB is a *bona fide* LC for DdMyo7.

### Calcium sensitivity of LC binding to the DdMyo7 HC

LC binding to the neck region of myosins is critical for proper motor function. Ca^2+^ binding to calmodulin LCs can result in either dissociation or tight binding depending on the myosin (7). The impact of Ca^2+^ on DdMyo7 LC binding was investigated by performing pull-downs either in the absence (–Ca^2+^) or presence (+Ca^2+^) of calcium. The DdMyo7 motor (shHMM-FD-GFP, Fig. 1) was isolated (GFP trap resin or α-GFP antibodies and protein A sepharose) from *talA* null cells coexpressing shHMM-FD and FL-CalB. The resulting pellet was examined for the presence of CalA and FL-CalB by immunoblotting with α-CaM and α-FLAG antibodies. Both LCs were present when DdMyo7 motor was precipitated in the absence of Ca^2+^ (Fig. 2*C*). However, CalA association with the DdMyo7 HC was abolished (>99%) in the presence of Ca^2+^ (Fig. 2*C*). Unexpectedly, CalB association was reduced by ∼75% of the –Ca^2+^ CalB signal, revealing that Ca^2+^ causes dissociation of a significant fraction of CalB, but unlike CalA, a small fraction remains bound to the HC (Fig. 2*C*). The significant loss of CalB in the presence of Ca^2+^ was not expected based on the notable differences in its EF hand sequences that would likely render CalB incompetent to bind Ca^2+^ (Fig. S1). However, the results here establish that Ca^2+^ does impact the LC-HC interaction of CalB. The observed loss of LC binding in the presence of Ca^2+^ suggests that DdMyo7 is negatively regulated by Ca^2+^.

### Characterization of dimerized DdMyo7 motors *in vivo*

**Figure 3:**
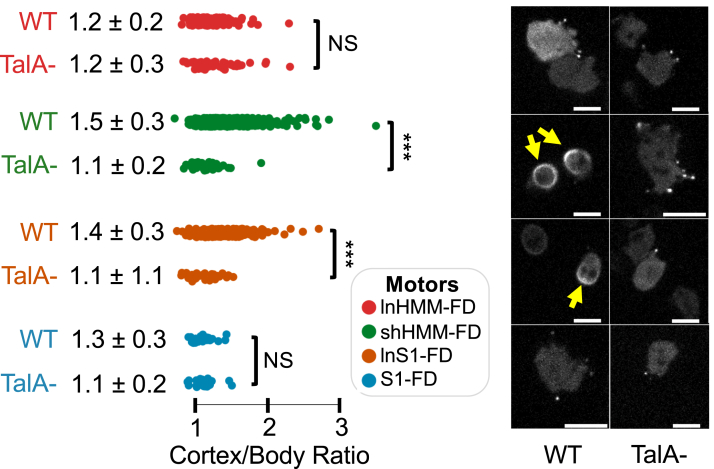
**Characterization of DdMyo7 motor localization.***Left*: quantification of cortex-to-body intensity ratio of each dimerized motor in WT or *talA* null cells. lnHMM-FD WT (N: 3, n: 178), lnHMM-FD TalA- (N: 5, n: 91), shHMM-FD WT (N: 3, n: 520), shHMM-FD TalA- (N: 4, n: 66), lnS1-FD WT (N: 3, n: 270), lnS1-FD TalA- (N: 3, n: 39), S1-FD WT (N: 4, n: 32), S1-FD TalA- (N: 2, n: 25). N = number of imaging sessions, n = number of cells (used for statistical analysis). One-way ANOVA with pairwise comparison (∗∗∗ *p* value: <0.001, NS: not significant). Mean and standard deviation reported. *Right*: representative WT (*left* column) and *talA* null (*right* column) cells expressing the motor fragments. *Yellow arrows* indicate shHMM-FD and lnS1-FD anchored by TalA at the cortex. The scale bars represent 10 μm. TalA, Talin A; FD, forced-dimer; lnHMM, long heavy meromyosin.

The DdMyo7 PLA is a region following the four IQ motifs that contains a single alpha-helix (SAH) domain, a candidate dimerization domain (cDD), and a proline-rich domain (Pro) (Fig. 1). The PLA region can play a role in determining step size, in particular the SAH domain (23), and potentially mediate DdMyo7 dimerization through a partner (cDD) (8). For example, a parallel forced-dimer (FD) of the DdMyo7 motor is targeted to filopodia tips and can partially rescue filopodia formation in DdMyo7 null cells (8) whereas a monomeric motor alone cannot (17). It was unclear how the PLA might impact motor stability, therefore, a set of DdMyo7 motors with different PLA lengths were dimerized using the Myo5A coiled-coil domain followed by a GCN4 leucine zipper motif (24) to identify a well-behaved dimerized motor fragment for characterization. These included the following: lnHMM-FD (full PLA); shHMM-FD (truncated Pro-rich); long subfragment-1 forced-dimer (lnS1-FD, SAH plus dimerization domain); and subfragment-1 forced-dimer (S1-FD, SAH only) (Fig. 1). Each of the motors was expressed in WT *Dictyostelium* cells to determine their localization, expression levels, and stability. lnHMM-FD and the motor alone S1-FD were largely cytosolic but also seen at the tips of filopodia (Fig. 3). The localization of the dimerized motors to the tip could be due to their ability to translocate along the filopodial actin core to the tip or the preferential recruitment of the motor to cortical regions of the cytoskeleton where VASP is actively organizing actin into parallel actin bundles, including the tip (25).

Surprisingly, two candidate motors, shHMM-FD and lnS1-FD, displayed a robust and sometimes asymmetric enrichment at the cortex (Fig. 3, right panels, yellow arrows). The asymmetric enrichment was quantified by determining the ratio of the motor in the cytosol (soluble) and the region of the cortex with the greatest motor-GFP signal using the filoTips notebook within the filoVision platform (26). A cortex/body ratio of 1.0 indicates that the measured intensities in the cytosol and cortex were equal, consistent with a highly soluble motor. However, a value greater than 1.0 indicates enrichment of the motor at the cortex with an average ratio of 1.2 being consistent with the normal enrichment of FL-DdMyo7 (25). In WT cells, shHMM-FD and lnS1-FD displayed a robust enrichment ratio of 1.5 ± 0.3 and 1.4 ± 0.3, respectively, while lnHMM-FD and S1-FD had ratios of 1.2 and 1.1, respectively, similar to that seen for FL-DdMyo7 (Fig. 3). The highly asymmetric localization of lnHMM- and S1-FD to the cortex suggested that these motors may be anchored there by a binding partner or possibly interaction with actin. TalA is the major binding partner for DdMyo7, and it has been implicated in regulating the turnover of the myosin at the leading edge of migrating cells (19, 20). The binding site for TalA was previously mapped to the PLA (Fig. 1), in a region present in all of the motor fragments except for S1-FD (20). Thus, the motor fragments were expressed in *talA* null cells (21). The cortical enrichment of shHMM-FD dropped to 1.1 ± 0.2 and that of lnS1-FD dropped to 1.1 ± 1.1, values similar to the cortical enrichment of lnHMM- and S1-FD in WT cells. This demonstrated that TalA was responsible for anchoring shHMM-FD and lnS1-FD to the actin-rich cortex (Fig. 3). The observed change in cortical targeting was not seen for either S1-FD or lnHMM-FD (Fig. 3), this was expected for S1-FD as it lacks the TalA binding site. However, it was surprising that lnHMM-FD displayed normal cortical localization in the presence or absence of TalA given that it contains the TalA binding region. This suggests that the C-terminal region of Pro1 could have a role in regulating the DdMyo7:TalA interaction in a way that promotes DdMyo7 dissociation from the cortex to maintain optimal levels of motor in this region.

A key to obtaining quantities of motor sufficient for purification is the establishment of cell lines that stably express the motor. The four different motors were each expressed in *talA* null cells, and the lines were assayed for expression stability using different growth conditions. shHMM-FD is the most stably expressed motor fragment based on having the lowest percentage of cells with aggregates or punctae after 2 weeks in culture (Fig. S4, *A* and *B*). Expression in cells grown in suspension for 48 h was also tested and, again, shHMM-FD had significantly fewer aggregates (Fig. S4*C*). Thus, shHMM-FD was chosen for purification and subsequent characterization *via* single molecule assay.

### shHMM-FD is a slow motor

**Figure 4:**
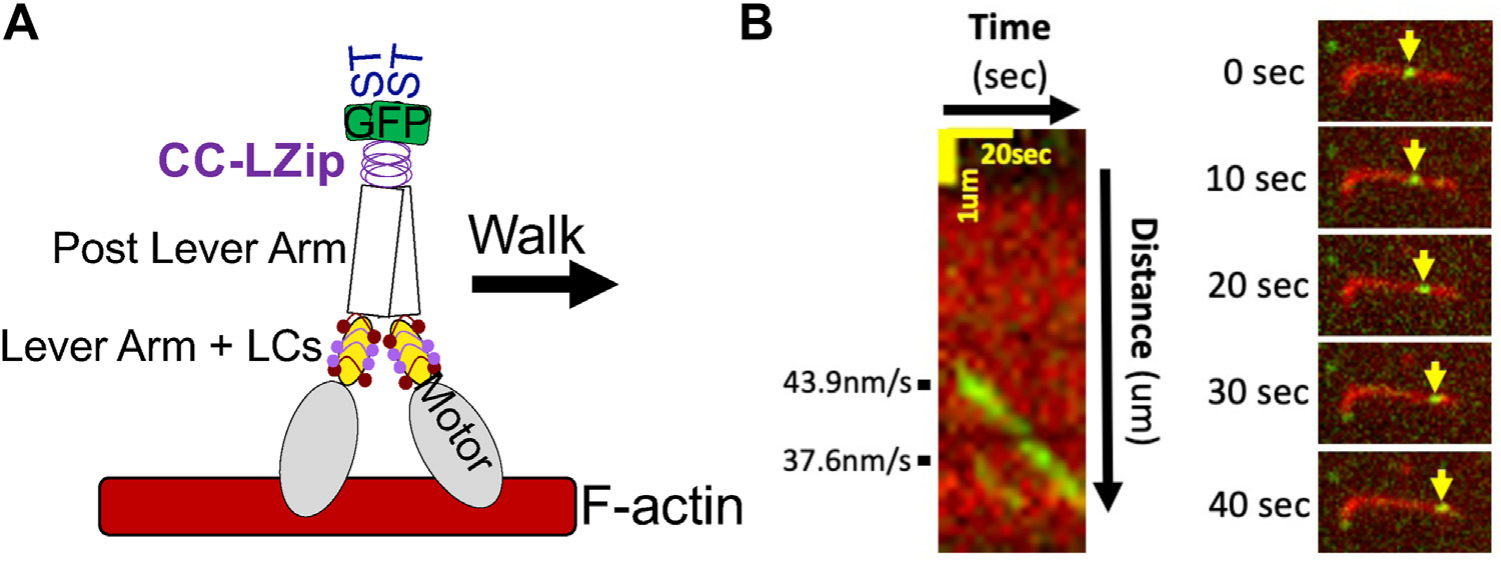
**TIRF-based in vitro motility assay.***A*, illustration of the *in vitro* motility assay. *B*, *Left*: kymograph of two representative motility events with velocities shown to the left. *Right*: sequential images of the upper event over 40 s (every 10 s) with *yellow arrows* tracking the individual shHMM-FD motor over time. FD, forced-dimer.

**Figure 5:**
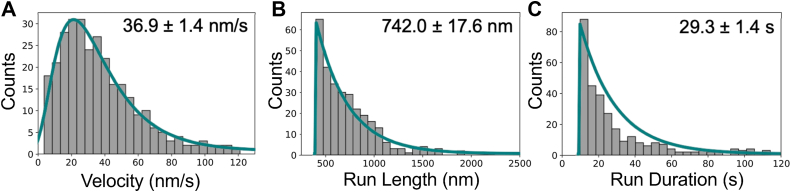
**Single molecule characterization of the DdMyo7 shHMM-FD motor**. Distribution of DdMyo7 shHMM-FD (*A*) velocity, (*B*) run length, and (*C*) run duration. N (motor purification): 3, N (chambers): 10, n (motility events): 414, mean ± S.E.M. reported. Data fit (*teal*) with either a log-normal (*A*) or exponential function (*B–C*). FD, forced-dimer.

A highly enriched fraction of shHMM-FD was prepared from a clarified *Dictyostelium* lysate *via* Strep-TactinXT four Flow affinity purification (Fig. S5, *A* and *B*). Note that excess CalA and CalB (Fig. S5*C*) was added at every step of the purification to ensure that the HC maintained a full complement of bound LCs. The motor activity of shHMM-FD was characterized by Total internal reflection fluorescence (TIRF) microscopy (27). Briefly, fluorescent biotinylated F-actin was attached to the surface of a motility chamber coated with streptavidin, a solution containing the shHMM-FD was flowed into the chamber, and then the movement of GFP labeled shHMM-FD along a single F-actin filament was observed (Fig. 4*A*). The shHMM-FD was first tested for its ability to bind and release from actin. A motor solution was flowed into an assay chamber lacking MgATP where it was seen to decorate F-actin anchored to the coverslip. shHMM-FD was then released from F-actin upon addition of MgATP, demonstrating a functional myosin motor (Fig. S6*A* and *B*). The motor properties of shHMM-FD were then measured under assay conditions similar to those used to characterize metazoan Myo7A and Myo10 motors (see Experimental procedures) (3, 9, 28). Three independent motor preps were analyzed over 10 separate chambers. GFP-tagged motors were clearly seen to move along actin filaments (see Fig. 4*B* and Supp Mov 1 and 2 for representative motility events). The velocity was determined using TrackMate (https://imagej.net/plugins/trackmate/) (29), revealing that shHMM-FD has an average velocity of 36.9 ± 1.4 nm/s, a run length of 742.0 ± 17.6 nm, and an average run duration of 29.3 ± 1.4 s on actin filaments (Fig. 5, *A*–*C*). These results demonstrate that shHMM-FD is a slow myosin capable of processive motility.

### Calcium inhibits DdMyo7 motor activity

**Figure 6:**
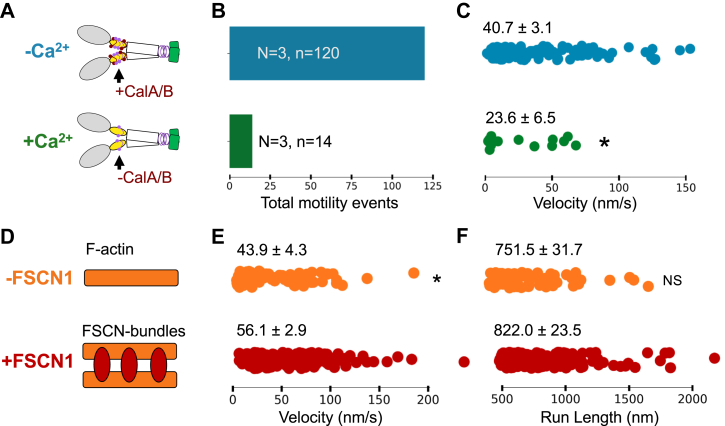
**DdMyo7 motility in the presence of calcium and on fascin-bundled actin.***A*, shHMM-FD motility minus (*blue*) and plus (*green*) calcium. *B*, total observed motility events minus (*blue*) and plus (*green*) calcium. N = motor purification and chamber number, n = individual motility events (used for statistical analysis) tracked over at least three frames. *C*, shHMM-FD velocity minus (N: 3, n: 120, *blue*) and plus (N: 3, n: 14, *green*) calcium. Two-sided Mann-Whitney *U* test, ∗ *p* value: 0.02, mean and S.E.M. reported. *D*, shHMM-FD motility on filamentous (*orange*) and FSCN-bundled (*red*) actin. *E*, shHMM-FD velocity on filamentous (N (motor purification): 2, N (chambers): 2, n (motility events): 74, *orange*) and FSCN-bundled actin (N (motor purification): 2, N (chambers): 3, n (motility events): 166, *red*). Two-sided student *t* test, *p* value: 0.02, mean and S.E.M. reported. *F*, shHMM-FD run length on filamentous (N: 2, n: 74, *orange*) and FSCN-bundled actin (N: 3, n: 166, *red*). Two-sided student *t* test, ∗ *p* value: 0.09, mean and S.E.M. reported. FD, forced-dimer; FSCN, fascin.

Myosin motor function is commonly regulated by bound LCs. Calcium can promote dissociation of the DdMyo7 LCs (Fig. 2*C*) suggesting that it can modulate motor activity. *In vitro* motility assays were performed with DdMyo7 in either the presence or absence of Ca^2+^ and the impact of Ca^2+^ on DdMyo7 motility observed (Fig. 6*A*). To prevent the loss of LC binding due to increased Ca^2+^, assays were performed in the presence of a large excess of purified CalA and CalB. The addition of Ca^2+^ to the motility assay caused a nearly nine-fold drop in the number of motility events from 120 in the −Ca^2+^ condition to just 14 events in the +Ca^2+^ condition over three independent experiments (Fig. 6*B*). The velocity of shHMM-FD also decreased, from 40.7 ± 3.1 nm/s -Ca^2+^ to 23.6 ± 6.5 nm/s + Ca^2+^ (Fig. 6*C*), demonstrating that DdMyo7 motor activity is indeed negatively regulated by the presence of calcium. Given that the DdMyo7 motor is essential for filopodia formation and motor activity is diminished in the presence of calcium, it stands to reason that Ca^2+^ levels could play a role in filopodia formation, in part by regulating the DdMyo7 motor.

### DdMyo7 motility is only slightly enhanced on FSCN bundles

Myo10 is an antiparallel dimer, and the two heads have an extended reach, enabling them to interact with adjacent actin filaments along a bundle. Consistent with this, its motor activity is optimal when the myosin walks on bundled F-actin (9)—the velocity of Myo10 moving on fascin (FSCN)-bundled actin filaments is 2x faster than what is observed for the motor moving on single filaments, and it has 2x longer run lengths on bundled actin (9). The finding that DdMyo7 is recruited to and activated by parallel bundles of actin at the cortex generated by VASP activity during the initiation and extension of filopodia formation suggested that its motor activity could also be enhanced on bundled filaments (25). The shHMM-FD motor activity on FSCN1-bundled actin filaments was analyzed (Fig. 6*D*), and its velocity was observed to increase only modestly when compared to single actin filaments, 43.9 ± 4.3 nm/s to 56.1 ± 2.9 nm/s, without a change in run length (Fig. 6, *E* and *F*). While significant, this relatively small increase in motility is not as substantial as seen for Myo10, suggesting that the DdMyo7 motor can fully function using any actin architecture. It should be noted, however, that the dimerized fragment used in this study is a parallel dimer, whereas Myo10 forms an antiparallel dimer. Since the DdMyo7 motor FD is seen at filopodia tips throughout elongation and partially rescues filopodia formation, it seems likely that the functional form of DdMyo7 is a parallel dimer; however, this has yet to be conclusively established.

## Discussion

Determining the motor properties of the *Dictyostelium* filopodial myosin is critical for understanding its mechanism of action during filopodia initiation and extension. *In vitro* motility assays of an FD of the DdMyo7 motor (shHMM) reveals that it is a slow processive motor that moves along single-actin filaments at ∼ 40 nm/sec with run lengths of ∼ 740 nm (Fig. 5). A slight increase in motor speed, ∼ 20% (56 nm/sec) is seen on FSCN-bundled actin filaments (Fig. 6, *D* and *E*), suggesting that DdMyo7 may be more efficient when moving along bundled filopodial actin filaments. The movement is much slower than the *Dictyostelium* filopodia elongation rate (∼300 nm/sec; (17)), so it is unlikely to serve as a fast transporter but instead may be better suited for a role in filopodia initiation, bringing actin filaments together at the cortex or acting as a tension-sensitive anchor. The tip localization of the motor suggests that it could also aid in anchoring the complex containing actin polymerases such as VASP and the *Dictyostelium* filopodial formin dDia2 to the filopodia tip.

### Filopodial myosins and intrafilopodial transport

The observed cotransport of Myo10 and VASP along the shaft of filopodia suggests that Myo10 may have a role in delivering the actin polymerase to the tip to support extension (12, 13). The motor moves up the filopodial actin core at a rate of ∼ 600 nm/sec and accumulates at the tip as extension proceeds (30), significantly faster than the rate of filopodia extension (33–80 nm/sec in HeLa cells) (14, 31, 32). In contrast, the velocity of shHMM-FD (∼40 nm/sec; Fig. 5) is much slower than the extension rate of *Dictyostelium* filopodia, ∼400 nm/sec (17) and DdMyo7 tip intensity levels remain constant during filopodia extension (8). The slow movement of DdMyo7 may not be sufficient to overcome retrograde flow. No information about retrograde flow within filopodia is available for *Dictyostelium*; however, it has been reported that the movement of conA-coated beads on the cell surface (an indirect read-out of retrograde flow) is ∼76 nm/sec (33), faster than the speed of shHMM-FD. These results suggest that DdMyo7 is unlikely to be able to transport cargo along the filopodial actin core to the tip during elongation and DdMyo7 in filopodia extension.

The notable difference between motor speeds and filopodia extension suggest that VASP activity has a significant role in determining the rate of filopodia extension in both mammalian cells and *Dictyostelium*. Consistent with this possibility, human VASP assembles actin at a significantly slower speed than *Dictyostelium* VASP *in vitro* (∼17 subunits/sec *versus* 74 subunits/sec; (34)), a difference in rates that are similar to that observed for filopodia extension velocities. However, VASP may not be the sole determinant of the elongation rate, a tip-localized motor may generate force at the tip to reduce membrane and allow G-actin monomer addition to the growing actin filaments ((14, 15) see below).

### Shared and distinct properties of Myo7 myosins

The slow processive motor properties of amoeboid DdMyo7 are quite similar to those metazoan Myo7 family members, *Drosophila* Myo7A (DmMyo7a) and human Myo7A. The shared motor properties of these related myosins reveals a remarkable degree of conservation across 600 million—1 billion years of independent evolution between Amoebozoa and Metazoa.

Myo7 motors move slowly and require partner-mediated dimerization for function—DmMyo7a moves along actin filaments at ∼ 8 nm/sec when dimerized by its partner M7BP (3), a FD of human MYO7A motor plus PLA (HsMyo7a) moves at 11 nm/sec and partner-dimerized full-length Myo7A moves at ∼ 8 to 22 nm/sec (35). However, Myo7 family members evolved to have different functions. DmMyo7A plays a role in maintaining the structure of denticles, embryonic actin-based protrusions reminiscent of stereocilia (36), and the auditory organ that also relies on bundled actin filaments for integrity (37). It has been shown to promote filopodia production in fly S2 cells when coexpressed with M7BP (3), but it is not yet known if it is required for filopodia production in flies. Human Myo7A and Myo7B are not filopodial myosins; they instead play critical roles in maintaining the organization of microvilli (MV) on intestinal epithelial cells and the hair bundle on auditory epithelial cells by anchoring cadherin-based linkages to the actin core of MV and stereocilia (SC), respectively (1, 38). The evolution of an anchoring function for Myo7 in metazoa likely coincided with the emergence of Myo10, a fast motor required for filopodia formation, in organisms that arose before the emergence of multicellularity (39).

### LC-mediated regulation of DdMyo7

Myosins are regulated by their associated LCs, typically CaM or calmodulin-family members, that bind to IQ motifs in the lever arm (7). The four LC binding IQ motifs of DdMyo7 are occupied by the *Dictyostelium* calmodulin, CalA, and a calmodulin-like protein, CalB, in a 1:2:2 ratio. The motor is negatively regulated by Ca^2+^ as binding of CalA and CalB is weakened in its presence, resulting in fewer motile events and significantly decreased speed (from 41 nm/sec to 24 nm/sec) (Figs. 2*C* and 6, *A–C*). The use of two or more related LCs is similar to mammalian Myo10 that binds CaM and a CaM-related LC, CLP. Human Myo7A has three different LCs (CaM, RLC, and CALML4) and Myo7B employs both calmodulin and CALML4 as LCs (35, 40, 41). The binding of both CalA and CalB is weakened in the presence of Ca^2+^, even though there are notable differences in three of the CalB EF hand sequences that are either missing critical residues for Ca^2+^ coordination and/or are slightly shorter than those of the *Dictyostelium* CaM, CalA (Fig. S1). This suggests that CalA binding and dissociation might play a role in modulating the interaction of CalB with its IQ domains. The reduced binding of both LCs to the DdMyo7 IQ region in the presence of Ca^2+^ would result in an inefficient power stroke likely due to decreased stiffness of the lever arm, reducing DdMyo7 activity and compromising filopodia formation. Interestingly, a *Dictyostelium* actin bundling proteins found in filopodia, actin-binding protein 34 (ABP34), is also negatively regulated by calcium (42). Together, these findings suggest that localized changes in the concentration of Ca^2+^ would play a critical role in regulating filopodia formation in *Dictyostelium*.

#### Filopodia formation driven by myosin motors

DdMyo7 and Myo10 are evolutionarily distant functional homologues. Both are essential for filopodia formation in cells (2, 4), but DdMyo7 moves at a significantly slower speed than mammalian Myo10 (40 nm/sec *versus* 300–600 nm/sec) (Figs 5; S7) (9). However, the two myosins do have common operational features, including the essential need for dimerization and the role of their C-terminal MF domains in modulating activity (2, 8, 17, 43). Dimerization is essential for DdMyo7 and Myo10 function as evidenced by the ability of dimerized motor domains alone, without any tail region, to promote filopodia formation and a loss of filopodia formation when the dimerization region is disrupted (8, 11, 43, 44). Although a dimerized motor alone is sufficient for filopodia formation, it is less efficient than the full-length motors, indicating that regions of the tail domains of both myosins make important contributions to their function (8, 44).

Filopodial myosin activity can be divided into two steps, initiation and elongation. During initiation, the dimerized filopodial myosins are recruited to the cortex where they coalesce into initiation punctae. The motors are likely organizing actin into nascent parallel bundles, alone or in cooperation with VASP or formin (1, 16, 45). Actin cross-linkers then rapidly stabilize the growing filopodial actin bundle. The DdMyo7 motors that cluster at the cortical membrane region become part of the filopodia tip where it may be in a complex with actin regulators that promote local actin polymerization for ongoing elongation. In contrast, Myo10 appears to change roles, switching from an organizer at the membrane to a transporter that moves along the filopodium to the tip. It is not yet clear if Myo10 is bringing materials required for ongoing elongation to the tip, such as supplying VASP as has been suggested based on their observed cotransport within growing filopodia (12, 13). Myo10 accumulates at the tip where it binds both the membrane through the three PH domains in its tail and the growing actin filaments. This would enable the motor to provide force to relieve membrane tension and allow the addition of actin monomers to the filament ends apposing the membrane (14). The observed behavior of DdMyo7 and Myo10 during the two critical stages of filopodia formation suggests that they may have similar roles during initiation but serve different functions during extension.

### Diverse myosin motors can promote filopodia formation and elongation

It is striking that myosins with quite different motor and kinetic properties can promote filopodia formation. A myosin that is not known to natively make filopodia can generate filopodia when ectopically expressed in cells. Human Myo3A is a slow motor that moves at ∼70 nm/sec and plays a role in the lengthening and widening of SC during the development of auditory hair cells. However, it promotes filopodia formation when ectopically expressed in COS7 cells (46). Myo3A chimeras consisting of the motor plus 2 IQ domains from different myosins that move with a range of speeds *in vitro*, from 290 nm/sec to 430 nm/sec, (Myo5A, Myo10, and Myo15), fused to the Myo3A tail promote extension of filopodia. Interestingly, as the speed of the chimeric motor increases, the rate of filopodia extension also increases (15). Ectopic expression of a membrane anchored Myo3A, Myo10, or Myo15 motor in HeLa cells can also promote elongation of existing filopodia (14). These chimeric myosins and membrane targeted motors localize to the tips of filopodia where it has been suggested that they play an important role in reducing membrane tension, allowing for addition of actin monomers and extension (14, 15). However, Myo3A chimeras of Myo1A, Myo7A, and NMIIA or membrane-docked motors of Myo1A, Myo5B, or Myo7B do not promote elongation and fail to localize to filopodia tips (14, 15). Differences in the ability of a myosin motor to promote filopodia formation and/or elongation or not could be due in part to differences in motor processivity, since Myo1A and NMIIA are nonprocessive motors, or to differences in the acto-myosin interface or the nature of tension generation by the various motors. Although it is curious that the membrane-targeted Myo7B motor cannot tip target even though the native full-length myosin does localize to the tips of MV. Further detailed characterization of how myosins interact with actin filaments and networks are needed to fully understand the underlying principles by which some myosin motors can promote filopodia formation while others cannot.

## Experimental procedures

### Cell culture

*Dictyostelium* cells were grown at 21 °C on plastic dishes in HL5 glucose medium (Formedium) supplemented with 10 kU/ml penicillin G and 10 μg/ml streptomycin sulfate (Sigma-Aldrich). Cells were transformed by electroporation of plasmids encoding different motor domain fragments (see Table S2 for details of plasmid construction and motor fragment details) (47) and selected, then the resulting cell lines (Table S3) maintained in 20 μg/ml G418 (neomycin resistance—Thermo Fisher Scientific) or 50 μg/ml hygromycin B (Gold Biotechnology). For motor purifications, cells were first grown on large 245 x 245 x 25 mm plates (Thermo Fisher Scientific, 240835) then moved into flasks of HL5. Motor expression dropped and aggregation increased while cells grew in suspension. This also became a problem if cell density exceeded 5 x 10^6^/ml. Thus, cell culture was tightly orchestrated to keep a density of around <2 x 10^6^/ml on large plates, and growth in suspension was limited to 36-48 h.

Growth of *talA* null cells expressing the different motor fragments was assayed by first screening a small culture of cells grown on plates over the course of 2 weeks by fluorescence microscopy. The culture was scored for the fraction of cells with bright green punctae or aggregates that indicated that the motor was unstable. The stability of motor expression in suspension grown cells was examined by growing cells on plates until confluent, transferring them to suspension for 48 h, and then measuring the percentage of puncta-free cells.

### Live-cell imaging

Live-cell microscopy was performed as previously described (17, 25). Cells in growth medium were plated in 35-mm glass dishes (MatTek P35G-1.5–14-C or CellVis, D35–10–1.5-N) at a density of ∼10^5^ cells/cm^2^ and allowed to adhere for 10 min. The attached cells were then rinsed twice in starvation buffer (16.8 mM sodium/potassium phosphate pH 6.4) and cells were imaged 45 to 75 min post starvation at 1 to 4 Hz on a spinning disk confocal (3i Marianas or Zeiss Axio Observer Z.1) with a 63 X/1.4NA Plan Apo oil-immersion objective. The temperature was maintained at 20 °C using a portable room air conditioner. Samples were illuminated with 50 mW lasers (488 nm or 561 nm) with Semrock filters for 488- or 561-nm excitation using a Yokogawa CSU-X1 spinning disk and captured with a Photometrics Evolve 512 EMCCD camera (final 0.212 micron pixel size). Subsequently, 4 to 6 Z sections of 0.28 to 0.5 microns were taken with a 50 to 250 ms exposure with 10 to 40% laser power. Cells were imaged for approximately 10 s and 10 to 20 fields of view were collected from each imaging dish consisting of 3 to 20 cells per field of view. Data sets consist of at least three independent experiments and two independently transformed cell lines.

### Filopodia number and cortical enrichment analysis

Filopodia number measurements and cortical enrichment analysis of dimerized DdMyo7 motors were performed using the filoTips notebook within the filoVision platform (26). One-way ANOVA with pairwise comparison was performed using the scipy.stats python library (∗∗∗ P-Val: <0.001, NS: not significant). Figures were generated with python libraries Seaborn and Matplotlib (48, 49).

### Identification of DdMyo7 LCs

The identification of DdMyo7 LCs was carried out by IP of lnHMM expressed in *talA* null cells (Table S2) followed by mass spectrometric analysis and Western blotting. Prior to pull downs, lnHMM was enriched by a slightly modified cytoprep method (50) to obtain a myosin-enriched cytoskeletal fraction. The cells were lysed in rigor conditions, centrifuged to obtain a cytoskeletal pellet and the pellet resuspended in an MgATP-supplemented release buffer which results in the release of bound myosins. A total of 2 x 10^9^ *Dictyostelium* cells expressing the tagged motor fragment were harvested, washed twice in rigor buffer (100 mM Tris–HCl pH 8.1, 5 mM EGTA, and 5 mM MgCl_2_), and then resuspended in 40 ml of rigor lysis buffer (rigor buffer, 0.5% Triton X-100 (Anatrace), 5U/μl CIP (New England Biolabs), and a protease inhibitor cocktail consisting of: 240 μg/ml Pefabloc, 37 μg/ml tosyl-L-lysyl-chloromethane hydrochloride, 35 μg/ml tosyl phenylalanyl chloromethyl ketone, 3.3 μg/ml E64, and 0.4 μg/ml ALLN). The lysate was centrifuged at 40k x *g* for 20 min to pellet the rigor cytoskeleton. The pellet was washed in low pH buffer (100 mM Pipes pH 6.8, 5 mM EGTA, and 5 mM MgCl_2_), centrifuged again at 40k x *g* for 10 min, and resuspended in buffer including 20 mM MgATP. This suspension was homogenized and centrifuged at 40k x *g* for 20 min. The release supernatant containing the tagged motor fragment, in addition to endogenous myosin II, was incubated with 200 μl of 50% anti-Flag bead slurry (Sigma-Aldrich, A2220) for 1 h. The anti-Flag resin was pelleted (640g, 5 min, 4 °C) and washed 3x in Flag buffer (25 mM Hepes pH 7.4, 300 mM NaCl, 10 mM MgCl_2_ and 5 mM EGTA, or 2 mM CaCl_2_). The motor was eluted by incubation with Flag buffer supplemented with 500 mM Arginine and 1 mg/ml Flag peptide (Sigma-Aldrich, F3290). L-arginine was added to enhance elution from FLAG resin (51). The eluate was separated on a 4 to 20% polyacrylamide TGX gel (Bio-Rad). The gel was stained with colloidal blue Coomassie (Invitrogen) to identify low molecular weight proteins that coprecipitate with the motor HC, then CalA was identified in the enriched motor fraction by an optimized immunoblotting method (52). The low molecular weight band predicted to be a LC (∼15–17 kD) was excised and identified by mass spectrometry analysis (Harvard-Taplin Mass Spectrometry Facility). The ratio of HC to LCs was determined by densitometry of gel bands. Gels of the FLAG IP pellet were stained with either colloidal blue Coomassie or Sypro Ruby (Invitrogen, visualized with Bio-Rad ChemiDoc) and band intensities of CalA, CalB, and the lnHMM HC measured. The intensity values were then normalized to their molecular weights, and the ratio of HC to LCs calculated.

### Motor pull downs in the presence of calcium

A total of 1 x 10^9^ *talA* null cells coexpressing shHMM-FD and Flag-CalB (Table S2) were lysed in 12 ml lysis buffer (50 mM Tricine pH 8.0, 150 mM NaCl, 5 mM MgCl_2_, 1% Triton X-100, 1 mM DTT, 20 mM MgATP, 2 mM EGTA, and the protease inhibitor cocktail) for 1 h. The cell lysate was centrifuged (4552*g*, 10 min, and 4 °C) and the GFP-motor immunoprecipitated from the supernatant using either a mixture of two anti-GFP monoclonal antibodies (12A6 and 4C9, Developmental Studies Hybridoma Bank) and protein A sepharose resin (Invitrogen, 101141), or with GFP-Trap beads (ProteinTech, gta). When protein A sepharose resin was used, the supernatant was precleared twice by incubating with a 100 μL bead slurry in IP wash buffer (50 mM Tricine pH 8.0, 150 mM NaCl, 5 mM MgCl_2_, 1 mM DTT, and 2 mM EGTA) for 15 min. The resin was removed by centrifugation (640*g*, 5 min, and 4 °C), the cleared lysate incubated with the anti-GFP antibodies for 1 h, then 200 μL protein A sepharose bead slurry was added, and the mixture was incubated at 4 °C with gentle rotation for 1 h. The resin was split in half and washed twice in either 5 ml IP wash buffer (+2 mM EGTA and no Ca2^+^ condition), or in 5 ml IP wash buffer + 2 mM CaCl_2_. The beads were collected, and the DdMyo7 motor was eluted with 30 μL ULSB (125 mM Tris pH 6.8, 20% glycerol, 4% SDS, 6M urea, and bromophenol blue). The same procedure was used for GFP-Trap beads except for preclearing the lysate.

### Line scan analysis along the length of filopodia

A custom Python script was made to extract and normalize the fluorescence intensities of DdMyo7-mCherry and GFP-CalB along the length of filopodia. A line is manually drawn starting ∼ 2 μm beyond a filopodium tip that goes through the tip along the shaft, ending adjacent to the cell membrane. This was repeated for 69 filopodia in three separate imaging experiments across 16 DdMyo7-null cells coexpressing DdMyo7-mCherry and GFP-CalB. Two separate plasmids (pDTi340 and pCalB4) were cotransformed to achieve coexpression; therefore, only cells expressing both DdMyo7-mCherry and GFP-CalB were selected for analysis. GFP-CalB expression was variable in coexpressing cells, with some cells expressing quite high levels of the fusion. Therefore, to avoid excessive cytosolic GFP signal that could impact analysis, cells with an extremely high GFP-CalB signal were not included in the analysis. DdMyo7 signal is the most intense, or peaks, at a filopodium tip (17); therefore, the DdMyo7-mCherry signal was used to align the filopodia by assigning the most intense DdMyo7 pixel along each line scan the filopodium tip (0 μm from filopodium tip), and the other pixels were assigned a distance from that point. GFP-CalB signal was also extracted along this line and plotted alongside DdMyo7-mCherry. The GFP-CalB and DdMyo7-mCherry pixel values along each filopodium were normalized to the most intense GFP-CalB or DdMyo7-mCherry value along the filopodium. At each distance from the tips of the filopodia, the mean intensity and S.E.M. was calculated.

### CalA and CalB purification

CalA and HIS-TEV (tobacco etch virus)-CalB expression plasmids (Table S3) were transformed into BL21-AI bacteria (Invitrogen). Cells were grown in LB supplemented with 100 μg/ml ampicillin (LB-AMP) overnight at 37 °C. Cells were diluted 1:50 in LB-AMP and grown at 37 °C until the absorbance at 600 nm reached 0.4 to 0.8. CalA and HIS-TEV-CalB expression was induced in LB-AMP supplemented with 0.5 mM IPTG and 0.2% arabinose for 4 h at 37 °C. The induced cells were pelleted, flash-frozen in liquid N_2_, and stored at −80 °C. CalA was purified using an established calmodulin purification method (53). Cells expressing HIS-TEV-CalB were lysed for 15 min in B-PER (Thermo Fisher Scientific) solution supplemented with 300 mM NaCl, 30 mM imidazole pH 7.5, 5 mM MgCl_2_, 50 mg/ml lysozyme, 2500 U/ml DNase, and an EDTA-free protease inhibitor tablet (Roche, 05892791001). The lysate was centrifuged at 21k x *g* for 20 min at 4 °C, and the supernatant passed through a 0.2 μm surfactant-free cellulose acetate filter (Thermo Fisher Scientific, 723–9920) before being injected onto a 5 ml His-Trap column (Cytiva, 17524802) connected to an AKTA Pure FPLC. HIS-TEV-CalB was eluted from the nickel column with HIS elution buffer (300 mM KCl and 500 mM imidazole pH 7.5) yielding ∼0.1 mg/ml of CalB. The eluate was diluted in HIS binding buffer (300 mM NaCl and 30 mM imidazole pH 7.5) and incubated overnight with 0.1 mg/ml TEV protease at 4 °C (TEV protease provided by Damien Rasmussen and Dr Nicholas Levinson) (54). The digested eluate was run back through the His-Trap column to capture the HIS-TEV protease and uncleaved tagged protein product while isolating CalB in the flowthrough. CalA and CalB were exchanged into 10 mM Hepes pH 7.2 by overnight dialysis, flash frozen in liquid N_2_, and stored at −80 °C.

### shHMM-FD purification

*talA* null cells expressing shHMM-FD were seeded on large plates (22 x 22 cm) and grown to confluence. The cells were resuspended from the plate and transferred into 2 L of media in a 6 L flask and shaken for no more than 2 days. Expression of shHMM-FD was validated *via* confocal microscopy (GFP signal) before proceeding with purification. A total of 2 x 10^9^ *Dictyostelium* cells were pelleted (1800*g*, 7 min, and 4 °C, J6 Beckman centrifuge) and washed twice with strep buffer (50 mM Tricine pH 8.0, 150 mM NaCl, 1 mM EGTA, and 5 mM MgCl_2_). Cells were resuspended in 10 ml strep buffer plus 1 mM DTT and protease inhibitors and excess purified LCs (2 μM CalA, 2 μM CalB), then Triton X-100 (Anatrace) was added to a final concentration of 1%. The lysate was incubated for 1 h on ice. Next, 20 mM MgATP was added to the lysate to release myosins from actin, and the mixture was centrifuged (20k x g 5 min) to pellet the cytoskeleton and large debris. The supernatant was supplemented with fresh protease inhibitors and passed through a 0.2 μm surfactant-free cellulose acetate filter (Thermo Fisher Scientific, 723–9920). The filtered supernatant was applied to a Strep-Tactin-XT four-Flow gravity column (IBA-Lifesciences, 2–5032–001). The column was washed with one volume of strep buffer supplemented with 20 mM MgATP, 5 mM biotin, and 1 mM DTT to remove actin and two nonspecific endogenously biotinylated proteins from the column (detected by immunoblotting with streptavidin-Alexa Fluor 680 – Invitrogen, S21378) (Fig. S5*B*), then washed with four column volumes of strep buffer supplemented with 1 mM DTT. The shHMM-FD was eluted using 10 ml strep elution buffer (50 mM Tricine pH 8.0, 150 mM NaCl, 1 mM EGTA, 5 mM MgCl_2_, 100 mM biotin, 2 μM CalA, 2 μM CalB, and 1 mM DTT) and motor-containing fractions identified by Sypro-stained SDS-PAGE gels and immunoblotting using an α-GFP antibody (BioLegend B34). The Strep-Tactin column eluate yielding ∼4 μg of HC was concentrated to ∼100 nM (Cytiva 30 kDa spin concentrator 6 ml, HC concentration estimated by comparing its SYPRO Ruby stain signal intensity to a known protein standard), subjected to a dead-head spin to remove inactive motors (27), exchanged into motility buffer (Zeba spin desalting column, Thermo Fisher Scientific; 20 mM Hepes pH 7.5, 20 mM KCl, 5 mM MgCl_2_, 0.5 mM EGTA, and 1 mM DTT), and supplemented with 0.5U/ml apyrase (Sigma-Aldrich, A6535) for >1 h on ice to remove trace amounts of ATP. Following use, the column was regenerated with 4 ml fresh 15 mM NaOH which was washed out with ∼15 ml strep buffer, stored in strep buffer at 4 ˚C and reused up to 3x for subsequent purifications.

### TIRF-based motility assays

Motility chambers were assembled as described (27). The plasma cleaned coverslips were treated with biotinylated PEG-Silane were used to prepare the <10 μl volume single molecule chambers (27). This resulted in a significant reduction of shHMM-FD sticking to the coverslip when attempting to image motility events. The chambers were washed with motility buffer (20 mM Hepes pH 7.5, 20 mM KCl, 5 mM MgCl_2_, 0.5 mM EGTA, and 1 mM DTT) then blocked with motility buffer supplemented with 1 mg/ml bovine serum albumin (1 min) and washed 3x with motility buffer. Motility buffer supplemented with 2 mg/ml neutravidin was added to the chamber (2 min) and the chamber then washed 3x with motility buffer. Next, 150 nM biotinylated tetramethylrhodamine-labeled rabbit skeletal F-actin was flowed into the chamber, and after 1 min the chamber was washed 3x with motility buffer. Motor solution (motility buffer + 10 nM shHMM-FD, 30 μM MgATP, 50 mM DTT, 1 μM CalA, 1 μM CalB, 2.5 mg/ml glucose, 100 μg/ml glucose oxidase, and 40 μg/ml catalase) was flowed into the chamber and the sample imaged immediately. Motility events were captured over 5 to 8 min videos at a frame interval of 3.5 s. For each motor purification, and before all motility experiments, shHMM-FD was tested in a separate chamber for its ability to undergo rigor and release by flowing the motor into the chamber without MgATP and imaging motor bound to F-actin. Motility buffer supplemented with 10 μM MgATP was introduced and release of the shHMM-FD from the filaments imaged, to demonstrate an active motor. Calcium's impact on motor function was determined by using an EGTA-free motor solution supplemented with 100 μM free calcium (calculated with MaxChelator) (55). FSCN-bundled actin was generated by incubating 50 μM tetramethylrhodamine-labeled F-actin with 9 μM FSCN1 (56) in motility buffer for > 4 h, then diluted to ∼1 μM F-actin prior to being introduced to the chamber.

ATP-sensitive actin binding of shHMM-FD was tested by flowing motor into the motility chamber containing surface-bound actin in the absence of ATP, and then adding 10 mM MgATP to the chamber. Filaments and bound motor were imaged using TIRF microscopy. Motor binding was quantified by first performing ImageJ (imagej.net/software/fiji/downloads) thresholding on the actin filament channel to obtain a binary mask of the filaments. This mask was then used to extract the mean fluorescence intensity of shHMM-FD-GFP along individual filaments using the shHMM-FD-GFP channel. shHMM-FD-GFP mean fluorescence intensity on actin filaments in the absence and presence of ATP was then quantified and compared.

### TIRF microscopy

TIRF motility assays were imaged using a Zeiss Axio Observer Z1 inverted microscope equipped with a motorized TIRF slider and Definite Focus. Motor-GFP and tetramethylrhodamine actin were imaged simultaneously and excited with 488 and 561 nm laser lines, respectively. Images were obtained with a 100x/1.46NA Alpha Plan-Apo objective. Images were collected with an EM-CCD Photometrics QuantEM camera using Axiovision 4.82 software. Motility events were captured over 5 to 8 min videos at a frame interval of 3.5 s.

### Motility data analysis

Motility data analysis was carried out to determine motor velocity and run lengths (27). Images collected using Axiovision 4.82 software were exported as .TIFF files. Standard ImageJ plugins and functions (Image Stabilizer cite, Bleach Correction cite, and Subtract Background cite) were used to stabilize, bleach-correct, and background subtract the movies. Next, TrackMate (https://imagej.net/plugins/trackmate/) (29) was used to detect and track motility events for the movie duration. Default TrackMate settings were used with a LoG detector to detect motor events with a blob diameter set to 1 μm and thresholded at 25. A simple LAP tracker (1 μm linking max distance and gap-closing max distance) was used with track filters in place, but not spot filters. Track filters included track displacement: >0.39 to remove motor/spots moving less than 3 pixels, and spots in tracks: >3 to remove tracks with less than three spots. The distribution of the raw TrackMate output was first inspected to detect obvious spurious events such as false motility events and stalled motors that started to move along actin tracks only to get stuck during a run (27). Two track duration filters were used to remove these events by defining a true event as one that must be tracked over at least three consecutive frames and a motor duration cutoff of 120 s to remove right-skewed boxplot outliers which were manually inspected and determined to be stalled motors. Student *t* tests were performed using the Researchpy python library. Mann-Whitney U tests were performed using the scipy.stats python library. The *p*-value cutoff for all tests were as follows: <0.05 ∗, <0.01 ∗∗, <0.001 ∗∗∗, <0.0001 ∗∗∗∗. Figures were generated using Seaborn and Matplotlib python libraries, and representative kymographs were generated using the kymograph builder plugin in Fiji/ImageJ (57) and analyzed using KymoButler (https://imagej.net/plugins/kymograph-builder) (58).

## Data availability

Data will be shared upon request to Margaret A. Titus (titus004@umn.ed).

## Supporting information

This article contains supporting information. Values measured in other studies are cited in Supporting Information (3, 9, 28) and references to plasmids used are cited in Table S2 (8, 9, 25, 59, 60, 61, 62).

## Conflict of interest

The authors declare that they have no conflicts of interest with the contents of this article.

## Supporting information

Table S3

Supporting Info

Supp Movie 1

Supp Movie 2

## References

[1] Houdusse A., Titus M.A. (2021). The many roles of myosins in filopodia, microvilli and stereocilia. Curr. Biol..

[2] Bohil A.B., Robertson B.W., Cheney R.E. (2006). Myosin-X is a molecular motor that functions in filopodia formation. PNAS.

[3] Liu R., Billington N., Yang Y., Bond C., Hong A., Siththanandan V. (2021). A binding protein regulates myosin-7a dimerization and actin bundle assembly. Nat. Commun..

[4] Tuxworth R.I., Weber I., Wessels D., Addicks G.C., Soll D.R., Gerisch G. (2001). A role for myosin VII in dynamic cell adhesion. Curr. Biol..

[5] Belyantseva I.A., Boger E.T., Friedman T.B. (2003). Myosin XVa localizes to the tips of inner ear sensory cell stereocilia and is essential for staircase formation of the hair bundle. PNAS.

[6] Probst F.J., Fridell R.A., Raphael Y., Saunders T.L., Wang A., Liang Y. (1998). Correction of deafness in *shaker-2* mice by an unconventional myosin in a BAC transgene. Science.

[7] Heissler S.M., Sellers J.R. (2014). Myosin light chains: teaching old dogs new tricks. Bioarchitecture.

[8] Arthur A.L., Songster L.D., Sirkia H., Bhattacharya A., Kikuti C., Borrega F.P. (2019). Optimized filopodia formation requires myosin tail domain cooperation. PNAS.

[9] Ropars V., Yang Z., Isabet T., Blanc F., Zhou K., Lin T. (2016). The myosin X motor is optimized for movement on actin bundles. Nat. Commun..

[10] Sakai T., Umeki N., Ikebe R., Ikebe M. (2011). Cargo binding activates myosin VIIA motor function in cells. PNAS.

[11] Tokuo H., Mabuchi K., Ikebe M. (2007). The motor activity of myosin-X promotes actin fiber convergence at the cell periphery to initiate filopodia formation. J. Cell Biol..

[12] Lin W.H., Hurley J.T., Raines A.N., Cheney R.E., Webb D.J. (2013). Myosin X and its motorless isoform differentially modulate dendritic spine development by regulating trafficking and retention of vasodilator-stimulated phosphoprotein. J. Cell Sci..

[13] Tokuo H., Ikebe M. (2004). Myosin X transports Mena/VASP to the tip of filopodia. Biochem. Biophys. Res. Commun..

[14] Fitz G.N., Weck M.L., Bodnya C., Perkins O.L., Tyska M.J. (2023). Protrusion growth driven by myosin-generated force. Dev. Cell.

[15] Cirilo J.A., Liao X., Perrin B.J., Yengo C.M. (2024). The dynamics of actin protrusions can be controlled by tip-localized myosin motors. J. Biol. Chem..

[16] Brown M.E., Bridgman P.C. (2003). Retrograde flow rate is increased in growth cones from myosin IIB knockout mice. J. Cell Sci..

[17] Petersen K.J., Goodson H.V., Arthur A.L., Luxton G.W., Houdusse A., Titus M.A. (2016). MyTH4-FERM myosins have an ancient and conserved role in filopod formation. PNAS.

[18] Sánchez-Soriano N., Gonçalves-Pimentel C., Beaven R., Haessler U., Ofner-Ziegenfuss L., Ballestrem C. (2010). *Drosophila* growth cones: a genetically tractable platform for the analysis of axonal growth dynamics. Dev. Neurobiol..

[19] Galdeen S.A., Stephens S., Thomas D.D., Titus M.A. (2007). Talin influences the dynamics of the myosin VII-membrane interaction. Mol. Biol. Cell.

[20] Tuxworth R.I., Stephens S., Ryan Z.C., Titus M.A. (2005). Identification of a myosin VII-talin complex. J. Biol. Chem..

[21] Niewöhner J., Weber I., Maniak M., Müller-Taubenberger A., Gerisch G. (1997). Talin-null cells of *Dictyostelium* are strongly defective in adhesion to particle and substrate surfaces and slightly impaired in cytokinesis. J. Cell Biol..

[22] Rösel D., Pûta F., Blahůsková A., Smýkal P., Folk P. (2000). Molecular characterization of a calmodulin-like *Dictyostelium* protein CalB. FEBS Lett..

[23] Baboolal T.G., Sakamoto T., Forgacs E., White H.D., Jackson S.M., Takagi Y. (2009). The SAH domain extends the functional length of the myosin lever. PNAS.

[24] Vavra K.C., Xia Y., Rock R.S. (2016). Competition between coiled-coil structures and the impact on Myosin-10 bundle selection. Biophys. J..

[25] Arthur A.L., Crawford A., Houdusse A., Titus M.A. (2021). VASP-mediated actin dynamics activate and recruit a filopodia myosin. Elife.

[26] Eddington C., Schwartz J.K., Titus M.A. (2024). filoVision - using deep learning and tip markers to automate filopodia analysis. J. Cell Sci..

[27] Tripathi A., Bond C., Sellers J.R., Billington N., Takagi Y. (2021). Myosin-specific adaptations of in vitro fluorescence microscopy-based motility assays. JOVE.

[28] Sato O., Komatsu S., Sakai T., Tsukasaki Y., Tanaka R., Mizutani T. (2017). Human myosin VIIa is a very slow processive motor protein on various cellular actin structures. J. Biol. Chem..

[29] Tinevez J.Y., Perry N., Schindelin J., Hoopes G.M., Reynolds G.D., Laplantine E. (2017). TrackMate: an open and extensible platform for single-particle tracking. Methods.

[30] Kerber M.L., Jacobs D.T., Campagnola L., Dunn B.D., Yin T., Sousa A.D. (2009). A novel form of motility in filopodia revealed by imaging myosin-X at the single-molecule level. Curr. Biol..

[31] Bornschlögl T., Romero S., Vestergaard C.L., Joanny J.F., Van Nhieu G.T., Bassereau P. (2013). Filopodial retraction force is generated by cortical actin dynamics and controlled by reversible tethering at the tip. PNAS.

[32] Masters T.A., Buss F. (2017). Filopodia formation and endosome clustering induced by mutant plus-end-directed myosin VI. PNAS.

[33] Jay P.Y., Elson E.L. (1992). Surface particle transport mechanism independent of myosin II in *Dictyostelium*. Nature.

[34] Breitsprecher D., Kiesewetter A.K., Linkner J., Urbanke C., Resch G.P., Small J.V. (2008). Clustering of VASP actively drives processive, WH2 domain-mediated actin filament elongation. EMBO J..

[35] Holló A., Billington N., Takagi Y., Kengyel A., Sellers J.R., Liu R. (2023). Molecular regulatory mechanism of human myosin-7a. J. Biol. Chem..

[36] Sallee J.L., Crawford J.M., Singh V., Kiehart D.P. (2021). Mutations in *Drosophila crinkled*/Myosin VIIA disrupt denticle morphogenesis. Dev. Biol..

[37] Todi S.V., Franke J.D., Kiehart D.P., Eberl D.F. (2005). Myosin VIIA defects, which underlie the Usher 1B syndrome in humans, lead to deafness in *Drosophila*. Curr. Biol..

[38] Weck M.L., Grega-Larson N.E., Tyska M.J. (2017). MyTH4-FERM myosins in the assembly and maintenance of actin-based protrusions. Curr. Opin. Cell Biol..

[39] Sebé-Pedrós A., Burkhardt P., Sánchez-Pons N., Fairclough S.R., Lang B.F., King N. (2013). Insights into the origin of metazoan filopodia and microvilli. Mol. Biol. Evol..

[40] Choi M.S., Graves M.J., Matoo S., Storad Z.A., El Sheikh Idris R.A., Weck M.L. (2020). The small EF-hand protein CALML4 functions as a critical myosin light chain within the intermicrovillar adhesion complex. J. Biol. Chem..

[41] Rogers M.S., Strehler E.E. (2001). The tumor-sensitive calmodulin-like protein is a specific light chain of human unconventional myosin X. J. Biol. Chem..

[42] Chung J.M., Kim H.U., Kim G.J., Jeoung D., Jung H.S. (2018). The actin bundling activity of actin bundling protein 34 is inhibited by calcium binding to the EF2. Biochem. Biophys. Res. Commun..

[43] Lu Q., Ye F., Wei Z., Wen Z., Zhang M. (2012). Antiparallel coiled-coil-mediated dimerization of myosin X. PNAS.

[44] Chen X., Arciola J.M., Lee Y.I., Wong P.H.P., Yin H., Tao Q. (2024). Myo10 tail is crucial for promoting long filopodia. J. Biol. Chem..

[45] He K., Sakai T., Tsukasaki Y., Watanabe T.M., Ikebe M. (2017). Myosin X is recruited to nascent focal adhesions at the leading edge and induces multi-cycle filopodial elongation. Sci. Rep..

[46] Raval M.H., Quintero O.A., Weck M.L., Unrath W.C., Gallagher J.W., Cui R. (2016). Impact of the motor and tail domains of class III myosins on regulating the formation and elongation of actin protrusions. J. Biol. Chem..

[47] Gaudet P., Fey P., Chisholm R. (2008). Transformation of *Dictyostelium* with plasmid DNA by electroporation. CSH Protoc..

[48] Hunter J.D. (2007). Matplotlib: a 2D graphics environment. Comput. Sci. Eng..

[49] Waskom M.L. (2021). Seaborn: statistical data visualization. J. Open Source Softw..

[50] Manstein D.J., Hunt D.M. (1995). Overexpression of myosin motor domains in *Dictyostelium*: screening of transformants and purification of the affinity tagged protein. J. Muscle Res. Cell Motil..

[51] Futatsumori-Sugai M., Abe R., Watanabe M., Kudou M., Yamamoto T., Ejima D. (2009). Utilization of Arg-elution method for FLAG-tag based chromatography. Protein Expr. Purif..

[52] Hulen D., Baron A., Salisbury J., Clarke M. (1991). Production and specificity of monoclonal antibodies against calmodulin from *Dictyostelium discoideum*. Cell Motil. Cytoskeleton..

[53] Gopalakrishna R., Anderson W.B. (1982). Ca2+-induced hydrophobic site on calmodulin: application for purification of calmodulin by phenyl-Sepharose affinity chromatography. Biochem. Biophys. Res. Commun..

[54] Majumdar A., Burban D.J., Muretta J.M., Thompson A.R., Engel T.A., Rasmussen D.M. (2021). Allostery governs Cdk2 activation and differential recognition of CDK inhibitors. Nat. Chem. Biol..

[55] Bers D.M., Patton C.W., Nuccitelli R. (1994). A practical guide to the preparation of Ca2+ buffers. Methods Cell Biol..

[56] Sherer L.A., Courtemanche N. (2022). Cooperative bundling by fascin generates actin structures with architectures that depend on filament length. Front. Cell Dev. Biol..

[57] Schindelin J., Arganda-Carreras I., Frise E., Kaynig V., Longair M., Pietzsch T. (2012). Fiji: an open-source platform for biological-image analysis. Nat. Methods.

[58] Jakobs M.A.H., Dimitracopoulos A., Franze K. (2019). KymoButler, a deep learning software for automated kymograph analysis. Elife.

[59] Knetsch M.L., Tsiavaliaris G., Zimmermann S., Rühl U., Manstein D.J. (2002). Expression vectors for studying cytoskeletal proteins in *Dictyostelium discoideum*. J. Muscle Res. Cell Motil..

[60] Levi S., Polyakov M., Egelhoff T.T. (2000). Green fluorescent protein and epitope tag fusion vectors for *Dictyostelium discoideum*. Plasmid.

[61] Veltman D.M., Akar G., Bosgraaf L., Van Haastert P.J. (2009). A new set of small, extrachromosomal expression vectors for *Dictyostelium discoideum*. Plasmid.

[62] Ulbricht B., Soldati T. (1999). Production of reagents and optimization of methods for studying calmodulin-binding proteins. Protein Expr. Purif..

