# Supplementary material for: The filopodial myosin DdMyo7 is a slow, calcium-regulated motor
Source: J Biol Chem. 2025 Mar 3;301(5):108371. doi: 10.1016/j.jbc.2025.108371 (PMC12125552; doi:10.1016/j.jbc.2025.108371)
Supplement: Table S2 [file mmc2.docx]

**Supporting Table 2. Generation of DdMyo7, CalA and CalB expression plasmids**

| **Plasmid** | **Expressed Protein/Region/PCR product** | **Template   or Backbone** | **Primers (5’ to 3’)/Ligations/Assemblies** |
| --- | --- | --- | --- |
| **pDTi381** | **Long HMM-GFP-FLAG   (aa 1-1115)** | pDXA-3H^59^ | pDTi375 4287 bp BamHI/XhoI insert ligated to pDXA-3H digested with BamHI/XhoI |
| pDTi375 | motor-Pro-GFP-FLAG | pSC-A* | Gibson assembly: pDTi349 minus 1497 bp BstEII/Xho 3’ fragment + Pro linker PCR prod + GFP-FLAG PCR prod |
|  | GFP-FLAG PCR prod | pTX-GFP^60^ | myi248G actgtttcaAGTAAAGGAGAAGAACTTTTCAC  myi251G gtaccgggccccccctcgagttatttatcatcatcatctttataatcTTTGTATAGTTCATCCATGCC |
|  | Pro linker PCR prod  (aa 612-1115) | pDTi111^19^ | myi233G cattagaattagaaaattggGTTACCCAATCCGTCATAC  myi247G tcctttactTGAAACAGTTGGTTGTGG |
| pDTi349 | motor-Pro (aa 1-1115) | pSC-A* | Ligation: pDTi324 minus 485 bp 3’ BglII/Xho fragment + pDTi323 1193 bp BglII/Xho fragment |
| pDTi323 | C-ter motor-Pro StrataClone (pSC-A)  (aa 681-1115) | pDTi111^19^ | myi11 ATTTGAAACTTGAGGAAC  myi201 ctcgagttaTGAAACAGTTGGTTGTGG |
| pDTi324 | motor-SAH StrataClone (pSC-A^*^) (aa 1-879) | pDTi111^19^ | myi65 cccGGATCCATGGAAGACGATGATACC  myi199 ctcgagttaTTTGAGCATTCTTTCGAGTGATG |
|  |  |  |  |
| **pDTi526** | **Long S1-Forced dimer-GFP-StrepTag (aa 1-974)** | pDM304^61^ | Ligation: pDM304-GFP-ST digested with BglII/SpeI + pDTi524 3825 bp BamHI/ SpeI fragment |
| pDTi524 | Long S1-Forced dimer   (aa 1-974) | pDTi498 | Q5 mutagenesis - deletion link CC F AGGTCTCAAGAAGCTGAAGATAG myi371 GATGCAGAAGCTTGTCTTTTAATAC |
| pDTi498 | HMM-Myo5CC-GCN4 StrataClone (in pSC-A*)  (aa 1-1115 no stop) | pDTi490 | myi65 cccGGATCCATGGAAGACGATGATACC GCN4 SpeR ACTAGTctccccgacaagcttctt |
| pDTi490 | GFP-HMM CC^8^ |  |  |
|  |  |  |  |
| **pDTi523** | **S1 Forced dimer-GFP-StrepTag (aa 1-860)** |  | Ligation: pDM304-GFP-ST digested with BglII/SpeI + pDTi521 3485 bp BamHI/ SpeI fragment |
| pDTi521 | S1-Forced dimer   (aa 1-860) | pDTi498 | Q5 mutagenesis - deletion link CC F AGGTCTCAAGAAGCTGAAGATAG myi370 GATGCTGATTTCTTTTCTTCCAATTTC |
|  |  |  |  |
| **pDTi514** | **Short HMM-Forced dimer-GFP-StrepTag   (aa 1-1021)** | pDM304^61^ | Ligation: pDM304-GFP-ST digested with BglII/SpeI + pDTi499 3967 bp BamHI/ SpeI fragment |
| pDTi499 | shHMM-Myo5CC-GCN4  (aa 1-1021) | pDTi498 | Q5 mutagenesis - deletion  CC F TCATCCCTCAAGAAGCTG  myi366 TTGTGATGTTGGAAGTGG |
|  |  |  |  |
| **pDTi510** | **Long HMM-Forced dimer-GFP-StrepTag   (aa 1-1115)** | pDM304^61^ | Ligation: pDM304-GFP-ST digested with BglII/SpeI + pDTi498 4244 bp BamHI/ SpeI fragment |
|  |  |  |  |
| **pDTi537** | **shHMM-Forced dimer-GFP-Srep Tag  + FLAG-CalB** | pDTi514 | Ligation: pDTi514 digested with NgoMIV + pCalB9 1052 bp NgoMIV fragment |
| **pDTi340**^24^ | DdMyo7-mCherry | pdM358^61^ |  |
|  |  |  |  |
| **Calmodulin Plasmids** | |  |  |
| pCalB9 | FLAG-CalB shuttle plasmid | pDM344^61^ | Ligation: pDM344 digested with BglII/Spe + pCalB6 480 bp BglII/Spe fragment |
| pCalB6 | FLAG-CalB StrataClone (in pSC-A*) | pCalB3 | calB9 atgGACTACAAGGACGACGACGACAAGGCAAAAGAAGATACTACTCAAGC  calB10 actagtTTAATTGAATGATTTGCTTG |
| **pCalB4** | **GFP-CalB *Dictyostelium* expression plasmid** | pDM317^61^ | Ligation: pDM317 digested with BglII/Spe + pCalB3 480 bp BglII/Spe fragment |
| pCalB3 | CalB StrataClone (in pSC-A*) | pCalB1 | calB3 actatacaagtccggactcagatctGCAAAAGAAGATACTACTCAAG  calB4 ttattaaataatttatttatttaactagTTAATTGAATGATTTGCTTGATTTAATAAC |
| **pCalB2-TV** | **His-TEV-CalB expression plasmid** | pCalB2 | Q5 mutagenesis to add TEV site  calB HisTEV F TTTCAGGGCGGAAGCCAGGATCCAATGGCA  calB HisTEV R ATACAGGTTTTCGTGGTGATGATGGTGATGG |
| pCalB2 | His-CalB bacterial expression plasmid | Ax2 cDNA | Gibson Assembly: RSFDUET-1 digested with BamHI/PstI + 492 CalB PCR product  calB1 accatcatcaccacagccaggatccaATGGCAAAAGAAGATACTAC  calB2 cgcaagcttgtcgacctgcagactagtTTAATTGAATGATTTGCTTGATTTAATAAC |
| pCalB1 | His-CalB/CalA bacterial expression plasmid | Ax2 cDNA | Gibson Assembly: pCalA digested with BamHI/PstI + 492 CalB PCR product  calB1 accatcatcaccacagccaggatccaATGGCAAAAGAAGATACTAC  calB2 cgcaagcttgtcgacctgcagactagtTTAATTGAATGATTTGCTTGATTTAATAAC |
| **pCalA** | **CalA bacterial expression plasmid** | pET-CAM^62^ | Gibson Assembly: RSFDUET-1 digested with NdeI/XhoI + 459 CalA PCR product  calA1 catATGGCATCACAAGAAAGTTTAAC  calA2 ctcgagTTAATTTCTAACAATCATCATTTTAAC |
| pET-CaM^62^ |  | pET-8c |  |
|  |  |  |  |
| **Base Cloning Plasmids** | |  |  |
| pDM304-GFP-ST | modified pDM304^61^ |  | Ligation: GGSGG GFP-ST SC 777 bp BglII/NheI insert + pDM304 digested with BglII/SpeI |
| GGSGG GFP-ST SC | GFP-Strep Tag StrataClone (in pSC-A^*^) | pDM1207^61^ | GFP ST F CAATTTGAAAAATAAGCTAGCAAGGGCGAA  GFP ST R TGGATGTGACCATCCGGATTTATATAATTCATCCATAC |

Lowercase letters indicate added restriction enzyme sites or overlapping sequences for Gibson assembly.

Cloning was carried out using enzymes, Q5 mutagenesis kit and HiFi Builder from New England Biolabs. TA-cloning of PCR products was performed using the Strataclone kit (Agilent). All PCR-generated sequences were verified by Sanger sequencing (Genewiz).

The pET-CaM plasmid was a kind gift from Thierry Soldati (U. Geneva)

^*^ Agilent StrataClone vector Tuxworth et al (2005); full-length *myoi* gene in pBluescript

8. Arthur, A. L., Songster, L. D., Sirkia, H., Bhattacharya, A., Kikuti, C., Borrega, F. P., Houdusse, A. and Titus, M. A. (2019) Optimized filopodia formation requires myosin tail domain cooperation. PNAS 116, 22196-22204

19. Tuxworth, R. I., Stephens, S., Ryan, Z. C. and Titus, M. A. (2005) Identification of a myosin VII/talin complex. J. Biol. Chem. 280, 26557-26564

24. Arthur, A. L., Crawford, A., Houdusse, A. and Titus, M. A. (2021) VASP-mediated actin dynamics activate and recruit a filopodia myosin. *Elife* **10**, 68082

59. Knetsch, M. L., Tsiavaliaris, G., Zimmermann, S., Rühl, U. and Manstein, D. J. (2002) Expression vectors for studying cytoskeletal proteins in *Dictyostelium discoideum*. J Muscle Res Cell Motil 23, 605-611

60. Levi, S., Polyakov, M. and Egelhoff, T. T. (2000) Green fluorescent protein and epitope tag fusion vectors for *Dictyostelium discoideum*. Plasmid 44, 231-238

61. Veltman, D. M., Akar, G., Bosgraaf, L. and Van Haastert, P. J. (2009) A new set of small, extrachromosomal expression vectors for Dictyostelium discoideum. Plasmid 61, 110-118

62. Ulbricht, B. and Soldati, T. (1999) Production of reagents and optimization of methods for studying calmodulin-binding proteins. Protein Expr Purif 15, 24-33
