# Supplementary material for: The filopodial myosin DdMyo7 is a slow, calcium-regulated motor
Source: J Biol Chem. 2025 Mar 3;301(5):108371. doi: 10.1016/j.jbc.2025.108371 (PMC12125552; doi:10.1016/j.jbc.2025.108371)
Supplement: Table S3 [file mmc3.docx]

**Supporting Table 3. Cell Lines**

| **Cell Line** | **Expressed Protein** | **Plasmid** | **Background** | **Reference** |
| --- | --- | --- | --- | --- |
| Ax2 | n/a | n/a | n/a | Dictybase.org |
| *talA* null  (HG1666) | n/a | n/a | n/a | ref. 20 |
| AA495-6 | lnHMM | pDTi381 | *talA* null | this paper |
| AC221 | shHMM-FD  + FLAG-CalB | pDTi537 | *talA* null | this paper |
| AC171 | FL-DdMyo7-mCherry  + GFP-CalB | pDTi340  + pCalB4 | Ax2 | this paper |
| CE64 | lnHMM-FD | pDTi510 | Ax2 | this paper |
| CE63 | shHMM-FD | pDTi514 | Ax2 | this paper |
| AC4 | lnS1-FD | pDTi526 | Ax2 | this paper |
| AC2 | S1-FD | pDTi523 | Ax2 | this paper |
| CE62 | lnHMM-FD | pDTi510 | *talA* null | this paper |
| CE61 | shHMM-FD | pDTi514 | *talA* null | this paper |
| AC5 | lnS1-FD | pDTi526 | *talA* null | this paper |
| AC2 | S1-FD | pDTi523 | *talA* null | this paper |
