# Supplementary material for: The filopodial myosin DdMyo7 is a slow, calcium-regulated motor
Source: J Biol Chem. 2025 Mar 3;301(5):108371. doi: 10.1016/j.jbc.2025.108371 (PMC12125552; doi:10.1016/j.jbc.2025.108371)

The filopodial myosin DdMyo7 is a slow, calcium regulated motor

Casey Eddington[1,2 ‡] & Margaret A. Titus[1,2*]

[1]Department of Genetics, Cell Biology, and Development, University of Minnesota, Minneapolis, MN, 55455, USA

[2]Graduate Program in Biochemistry, Molecular Biology, and Biophysics, University of Minnesota, Minneapolis, MN, 55455, USA

[‡] present address: Center for Learning Health System Sciences, University of Minnesota Medical School, Minneapolis, MN, 55455, USA

*To whom correspondence should be addressed e-mail: titus004@umn.edu

Supporting Information

Figure S1. Alignment of *Dictyostelium* CalA and CalB

Clustal Omega alignment of *Dictyostelium* CalA (UNIPROT P02599) and CalB (UNIPROT O00897) with human Calm1 (UNIPROT P0DP23) and Calm4 (UNIPROT Q96GE6-1, short isoform starting at amino acid 44). The four EF hands are highlighted with red boxes, residues known to be critical for Ca^{2+} coordination are highlighted in bold red and the boundary between the N- and C-terminal halves of the calmodulins is indicated by the green highlighted residues. Plain bolded residues in Calm4 and CalB EF hands have a different charge from canonical residues in that position. The ***** indicates amino acid identity and **.** or **:** indicates amino acid conservation. Note that EF2 and EF4 in CalB are predicted to be reduced in size as each is missing one amino acid.

Figure S2. Variable migration of CalB in SDS-PAGE gels.

Representative Sypro-stained 4-20% SDS-PAGE gradient gels illustrating an inconsistency in CalB electrophoretic mobility relative to CalA. Left – CalA and CalB running at slightly different molecular weights. Right – CalA and CalB running together.

Figure S3. CalB localization to filopodia tips. (A) Spinning disc confocal micrograph of

DdMyo7-mCherry (magenta) co-expressed with GFP-CalB (green). Yellow arrows indicate co-localization of DdMyo7 and CalB in filopodia tips. **(B)** Mean mCherry or GFP intensity along the length of filopodia extended by DdMyo7-null cells co-expressing DdMyo7-mCherry and GFP-CalB. Dark line indicates the mean intensity at each distance from the filopodium tip (x-axis) and the lighter shading represents the S.E.M. at each distance from the filopodium tip. Imaging experiments: 3, Cells: 16, Filopodia: 69.

Figure S4. PLA length impacts motor stability

(A) Representative *talA* null cells expressing dimerized motor fragments two weeks post-transformation. Yellow arrows indicate motor puncta/aggregation. **(B)** Percentage of puncta-free cells after two weeks of growth. Bar plot colors correspond with motor label colors. Left to right: lnHMM-FD (N: 3, n: 467), shHMM-FD (N: 3, n: 572), and lnS1-FD (N: 3, n: 1001). **(C)** Percentage of puncta-free cells after growth for 48 hrs in suspension. Left to right: lnHMM-FD (N: 3, n: 118), shHMM-FD (N: 3, n: 106).

Figure S5. Purification of the DdMyo7 heavy and light chains.

(A) shHMM-FD concentrated eluate supplemented with 2 µM excess CalA and CalB to ensure the purified heavy chain has its full light chain complement and help prevent aggregation of the motor. **(B)** Western blot (top) and Coomassie R-250 (bottom) analysis of shHMM-FD purification steps. The blot was probed with α-GFP (shHMM-FD) and streptavidin-Alexa Fluor 680 (Biotin-P*). Shown are the whole cell lysate (WCL), lysate supernatant after pelleting the cytoskeleton (SUP), the filtered lysate applied to column (Input), the column flow-through (Flow), column wash (Wash), example eluted fraction (Eluate), concentrated pool of eluted motor (Conc), and the final, desalted motor fraction (Desalt). Note that two high MW, non-specific biotinylated background proteins in the lysate are removed from the wash with the addition of 5 mM biotin. All purification steps included excess LCs and these are seen prominently in the final, enriched motor fraction. **(C)** Purified bacterially expressed CalA and CalB.

Figure S6. shHMM-FD strongly binds actin in the absence of ATP and weakly binds actin in the presence of ATP. (A) Representative images of shHMM-FD (green) in a strong F-actin (magenta) binding state in the absence of ATP (left), and shHMM-FD in a weak actin binding state in the presence of ATP (right). **(B)** Quantification of shHMM-FD-GFP mean fluorescence intensity on actin filaments. ATP- (Images: 3, filaments: 243, mean ± S.E.M.: 1,337.9 ± 45.4). ATP+ (Images: 3, filaments: 264, mean ± S.E.M.: 356.6 ± 4.9). Student's t-test P-val: < 0.0001

(individual filaments used for statistical analysis).

Figure S7. DdMyo7 motor activity comparison to HsMyo7A, DmMyo7A, and Myo10
(A) Comparison of DdMyo7 average velocity (blue) to other slow Myo7s, HsMyo7A and DmMyo7A (red), and the fast Myo10 motor (green) on filamentous and FSCN-bundled actin. **(B)** Comparison of DdMyo7 average run length (blue) to the run lengths of HsMyo7A and DmMyo7A (red), and Myo10 (green) on filamentous and FSCN-bundled actin [3, 9, 27].

Table S1. Raw mass spectrometry data from analysis of the low molecular weight band associated with the DdMyo7 motor.
List of the top ten targets resulting from excising the lower MW LC band and analyzing by mass spectrometry, leading to the revelation that calB is a DdMyo7 light chain.

Table S2. Generation of DdMyo7, CalA and CalB expression plasmids
Summary of DdMyo7, CalA and CalB expression plasmids and how they were generated [8, 9, 24, 59-62].

Table S3. Cell lines.
Cell lines used in this study.

Supplemental Video 1. Example of a single motor (green) moving on an actin filament (red). Yellow arrowhead illustrates movement along the filament.

Supplemental Video 2. Example of a full field of view in the TIRF motility assay showing single motors (green) moving on individual actin filaments (red). Several independent events are highlighted by the yellow tracking arrows.

SUPPORTING FIGURE 1

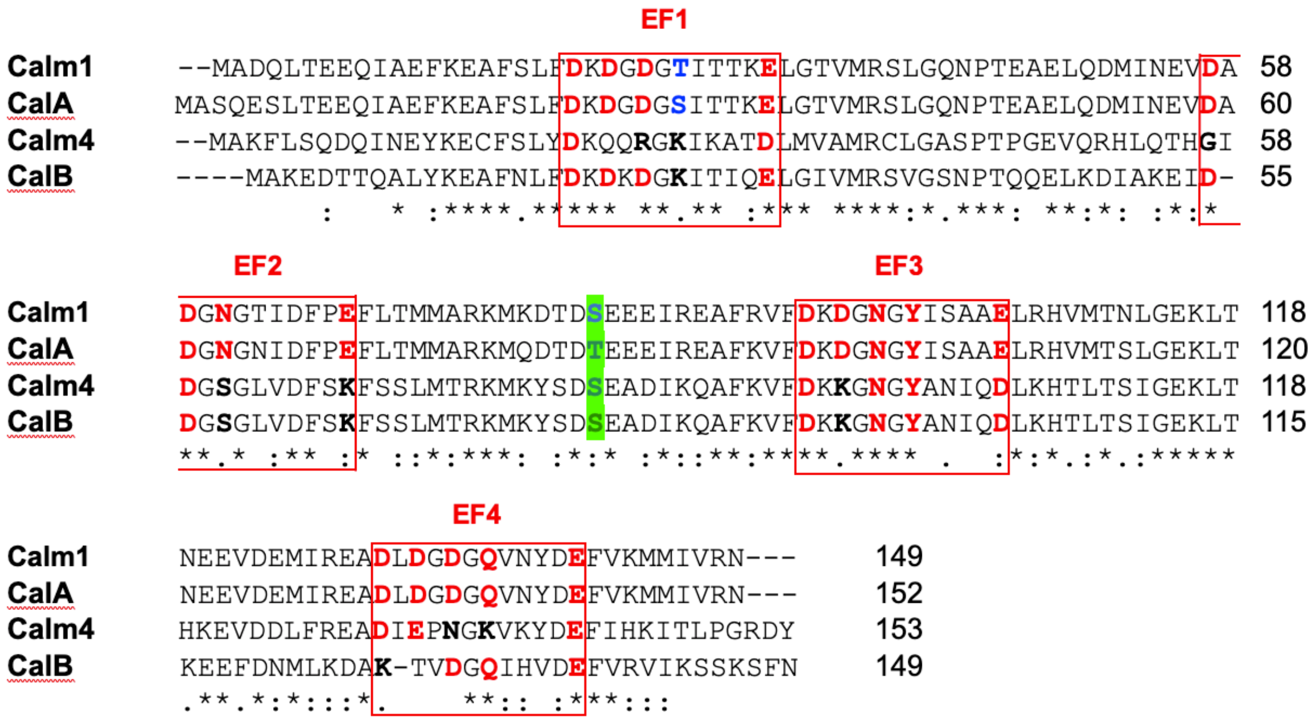

SUPPORTING FIGURE 2

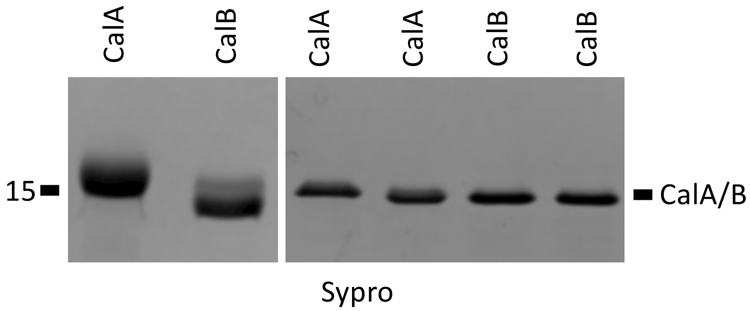

SUPPORTING FIGURE 3

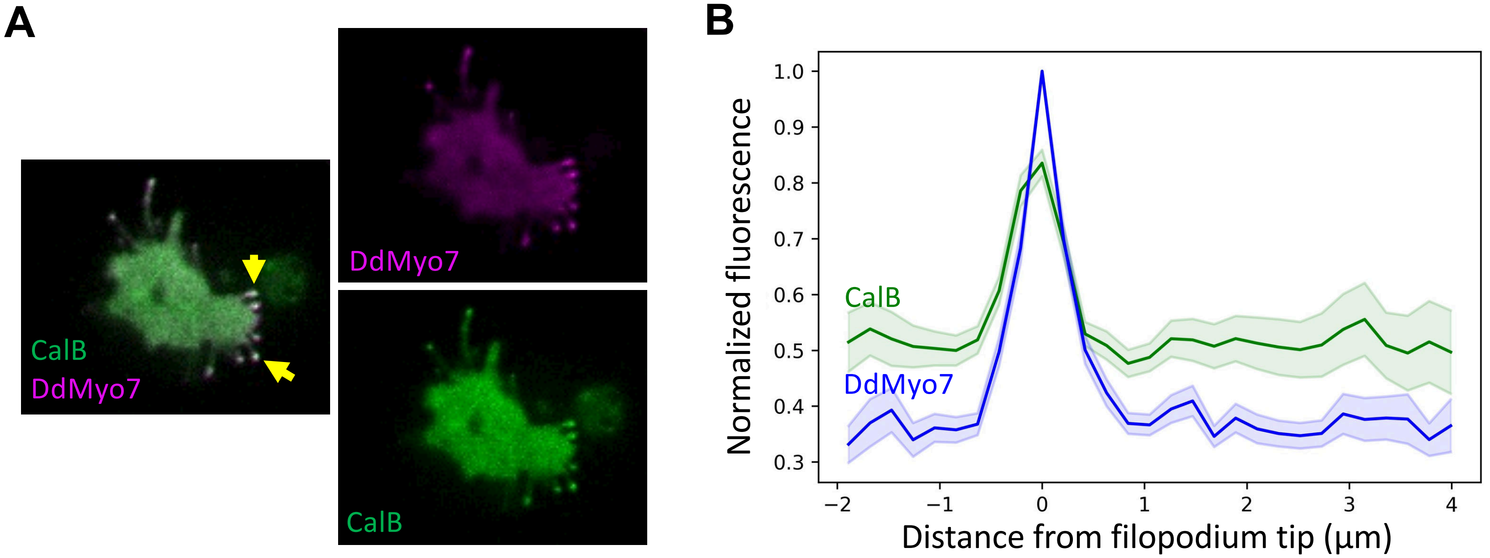

SUPPORTING FIGURE 4

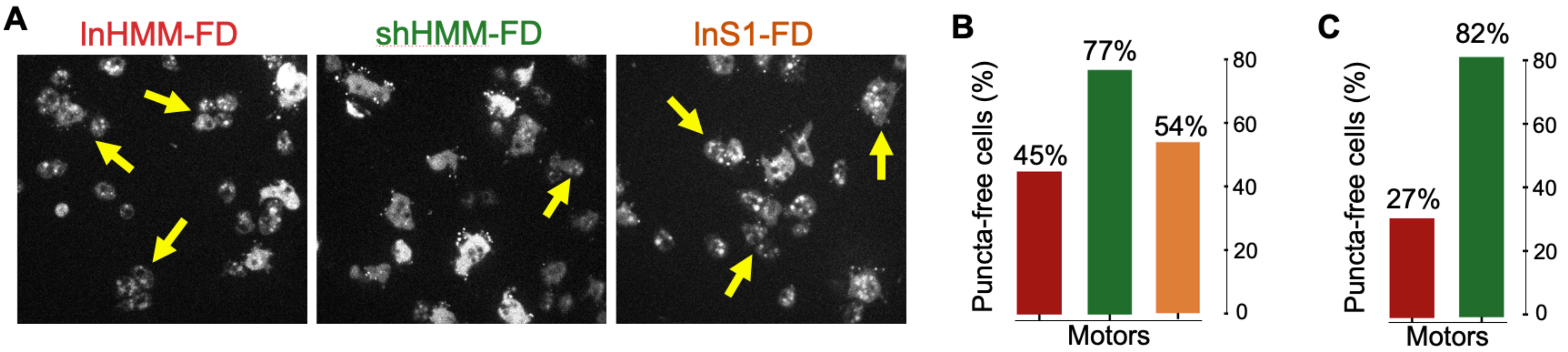

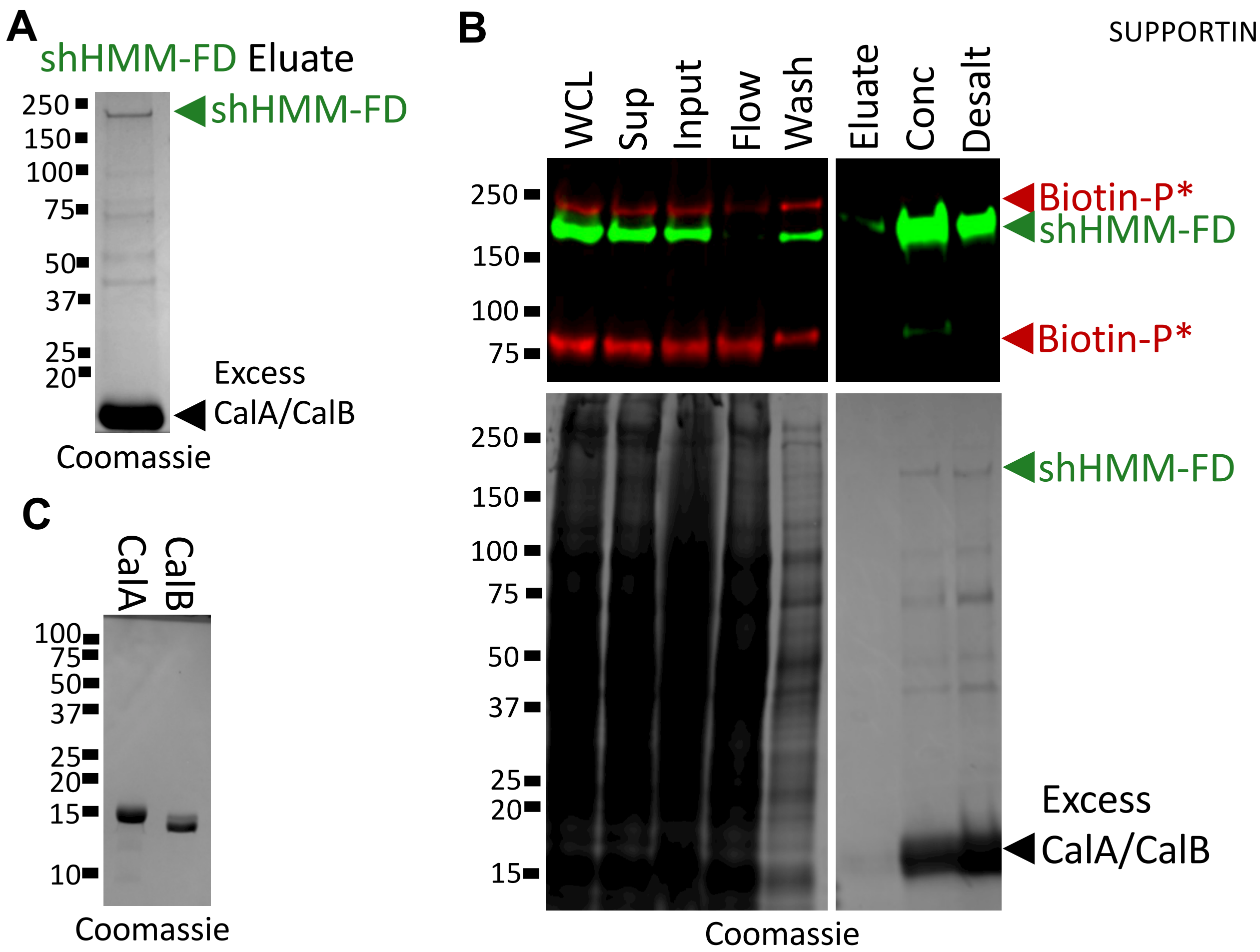
A
shHMM-FD Eluate
250
150
100
75
50
37
25
20
shHMM-FD
Excess
CalA/CalB
Coomassie
B
WCL
Sup
Input
Flow
Wash
Eluate
Conc
Desalt
250
150
100
75
Biotin-P*
shHMM-FD
Biotin-P*
250
150
100
75
50
37
25
20
15
shHMM-FD
Excess
CalA/CalB
Coomassie
C
CalA
CalB
100
75
50
37
25
20
15
10
Coomassie

shHMM-FD GFP signal on actin filaments +/- ATP

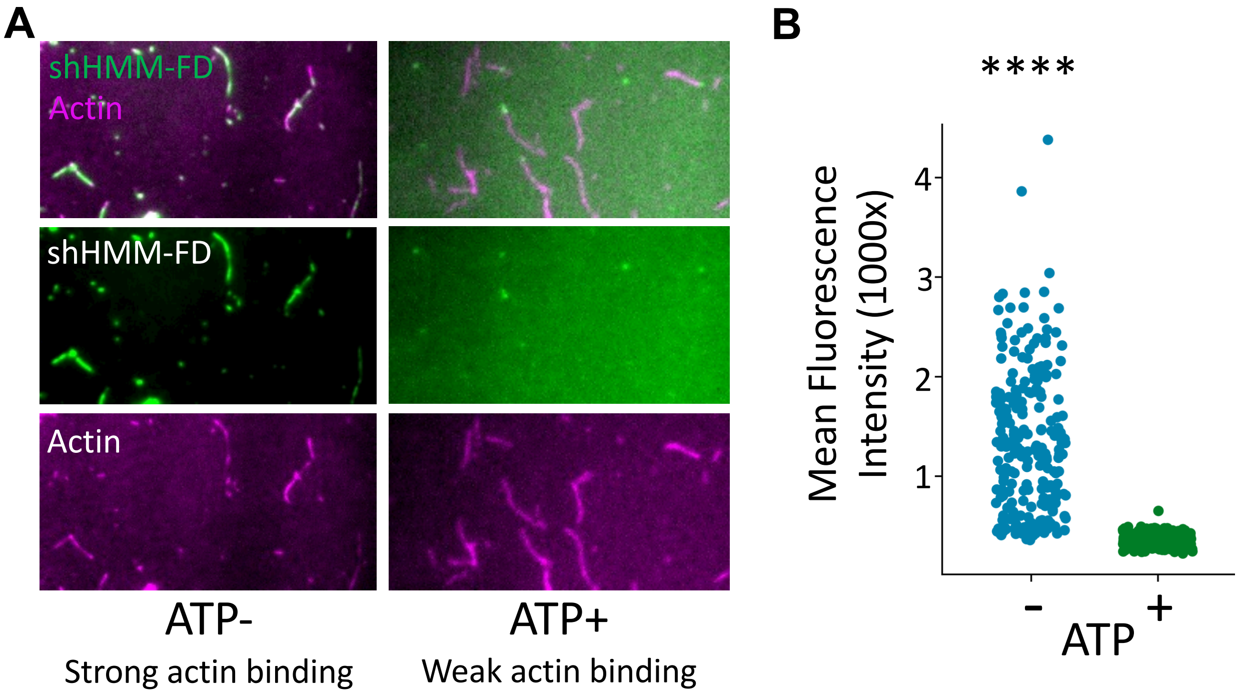

SUPPORTING FIGURE 7

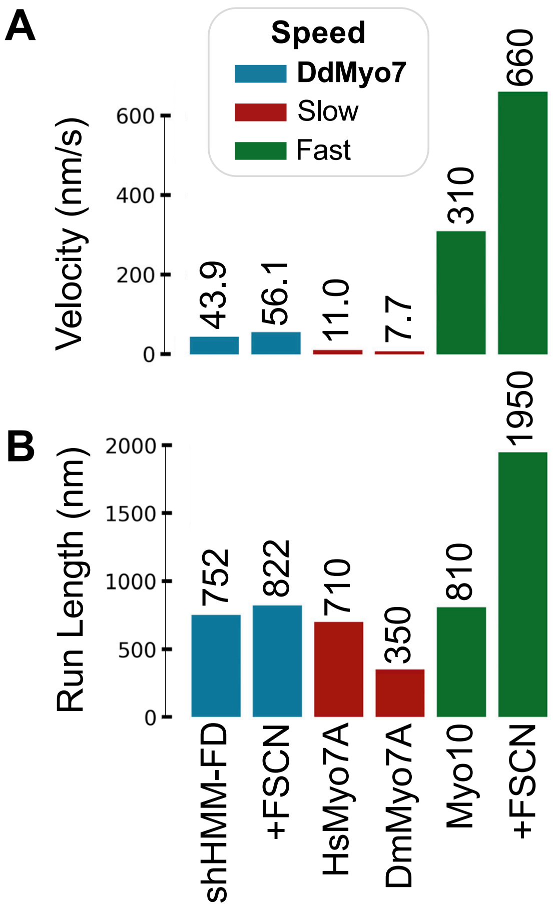

Supplement: Supporting Info [file mmc4.pdf]
